# Hamiltonian Computational Chemistry: Geometrical Structures in Chemical Dynamics and Kinetics

**DOI:** 10.3390/e26050399

**Published:** 2024-04-30

**Authors:** Stavros C. Farantos

**Affiliations:** 1 Department of Chemistry, University of Crete, GR-700 13 Heraklion, Greece; farantos@uoc.gr or farantos@iesl.forth.gr; 2 Institute of Electronic Structure and Laser, FORTH, GR-711 10 Heraklion, Greece

**Keywords:** Hamiltonian classical mechanics, geometrical quantum mechanics, Hamiltonian thermodynamics, chemical kinetics, differential geometry

## Abstract

The common geometrical (symplectic) structures of classical mechanics, quantum mechanics, and classical thermodynamics are unveiled with three pictures. These cardinal theories, mainly at the non-relativistic approximation, are the cornerstones for studying chemical dynamics and chemical kinetics. Working in extended phase spaces, we show that the physical states of integrable dynamical systems are depicted by Lagrangian submanifolds embedded in phase space. Observable quantities are calculated by properly transforming the extended phase space onto a reduced space, and trajectories are integrated by solving Hamilton’s equations of motion. After defining a Riemannian metric, we can also estimate the length between two states. Local constants of motion are investigated by integrating Jacobi fields and solving the variational linear equations. Diagonalizing the symplectic fundamental matrix, eigenvalues equal to one reveal the number of constants of motion. For conservative systems, geometrical quantum mechanics has proved that solving the Schrödinger equation in extended Hilbert space, which incorporates the quantum phase, is equivalent to solving Hamilton’s equations in the projective Hilbert space. In classical thermodynamics, we take entropy and energy as canonical variables to construct the extended phase space and to represent the Lagrangian submanifold. Hamilton’s and variational equations are written and solved in the same fashion as in classical mechanics. Solvers based on high-order finite differences for numerically solving Hamilton’s, variational, and Schrödinger equations are described. Employing the Hénon–Heiles two-dimensional nonlinear model, representative results for time-dependent, quantum, and dissipative macroscopic systems are shown to illustrate concepts and methods. High-order finite-difference algorithms, despite their accuracy in low-dimensional systems, require substantial computer resources when they are applied to systems with many degrees of freedom, such as polyatomic molecules. We discuss recent research progress in employing Hamiltonian neural networks for solving Hamilton’s equations. It turns out that Hamiltonian geometry, shared with all physical theories, yields the necessary and sufficient conditions for the mutual assistance of humans and machines in deep-learning processes.

## 1. Introduction

Chemistry is now deeply rooted in the two fundamental physical theories, quantum and classical mechanics. Quantum chemistry and molecular dynamics computer programs are indispensable devices in almost all kinds of chemical research. Thermodynamics, also a vital theory for chemistry that lacked a genuine mathematical foundation for years, has acquired the necessary mathematical framework in the last two decades, which brings it to the same level as classical and quantum mechanics. On the other hand, the advancements in mathematics that occurred in the 20th century in the fields of differential geometry and topology have revealed common geometrical structures in all physical theories. These discoveries provide a deeper understanding of the physical theories per se and pave the way for their application in other scientific fields, such as chemistry.

The purpose of this article is to render an introductory and graphical presentation of common geometrical structures of the principal physical theories and the consequences they may have in chemistry, especially via their numerical applications. Specifically, after almost two centuries of evolution of Hamiltonian theory, a modern geometrical description emerged, and significant theorems and techniques for locating invariant structures in phase space, and thus constants of motion, have been found [1,2,3]. Chemical dynamics and spectroscopy have tremendously benefited from applying these methods to comprehend the behaviors of highly excited molecules [4,5].

It is essential to underline that the stage of action for molecular dynamics, within a classical mechanical approach, is the phase space and its tangent bundle, which means that both generalized coordinates and conjugate momenta should be taken into account. We also emphasize that in the 1980s, it was found that quantum and classical mechanics share some common geometrical properties, which explain similarities as well as differences. It was shown that instead of the usual algebraic linear quantum theory formulated either in the Schrödinger or Heisenberg picture, we can take the quantum analog of phase space to be the projective Hilbert space of the extended Hilbert space [6,7,8].

Earlier, in the 1970s, it was recognized that the mathematical framework for thermodynamics is the contact geometry [9,10] of the physical states embedded in an odd-dimensional state space [11,12,13,14,15,16,17]. However, at the beginning of the twenty-first century, Balian and Valentin [18] made a significant contribution by publishing a Hamiltonian theory of thermodynamics in an extended phase space. Based on Callen’s [19] formulation of thermodynamics, they produced a Hamiltonian theory for reversible and irreversible processes equivalent to classical mechanics. They studied homogeneous Hamiltonians with generalized coordinates, the complete set of extensive properties (i.e., entropy, internal energy, volume, particle numbers, etc.) and conjugate momenta proportional to intensive properties (i.e., temperature, pressure, chemical potential, etc.), either in the entropy or energy representation. Gibbs’s fundamental equation was employed to describe the physical state manifold. This work triggered numerous studies, the results of which have revealed several geometrical properties common to classical and quantum mechanics [20,21,22,23,24].

It turns out that the mathematical abstraction and generalization of geometrical Hamiltonian theory in extended phase space lead to a common computational platform for working in both microscopic and macroscopic worlds. The aim of the present article is to highlight that Hamiltonian theories share some common geometrical properties in extended phase space with classical mechanics, quantum mechanics, and thermodynamics, which manifest the foundations for formulating and comprehending chemical dynamics and chemical kinetics.

In Section 2, we present, with the help of two pictures, the Hamiltonian theories of classical and quantum mechanics. Similarly, in Section 3, we discuss Hamiltonian thermodynamics in contemporary mathematical language that unveils the geometrical properties of this theory. In Section 4, we describe numerical algorithms for solving Hamilton’s equations of motion and variational equations, common to the three principal theories. We mainly focus on high-order finite-difference (FD) methods and their relation to pseudospectral methods (PS) [25]. Furthermore, to illustrate the mathematical concepts introduced in the previous sections, we have performed numerical calculations with a rather simple two-dimensional nonlinear model, that of Hénon–Heiles [26]. We deduce that high-order finite-difference methods based on the Lagrangian interpolation polynomials are appropriate for solving initial value problems, as well as partial differential equations, such as the Schrödinger equation, necessary in chemical theories. Finally, the conclusions are summarized in Section 5, where recent research on Hamiltonian neural networks (HNN) is discussed. Proofs for some equations, tables that summarize results in Hamiltonian thermodynamics, and an example of formulating a chemical kinetic model with thermodynamic Hamiltonian theory are presented in Appendix A.

## 2. Geometrical Structures in Chemical Dynamics

Molecules are sets of $N_{n}$ nuclei and $N_{e}$ electrons interacting with Coulomb forces. Usually, their quantum mechanical treatment is obtained by solving the Schrödinger equation in the Born–Oppenheimer approximation [27] that separates the electronic from the nuclear motion. The electronic energies are calculated by freezing the nuclei at specific configurations, which produces the adiabatic potential energy surface for each electronic state.

These molecular potentials, named also ***Potential Energy Surfaces***, are employed to solve the nuclear equations of motion either in quantum mechanics or in classical mechanics. It is, thus, important to investigate common geometrical structures in the foundations of the two basic theories of physics, which in turn may assist in the numerical solutions of the corresponding equations of motion. In the following two subsections, we examine the topological and geometrical properties of classical and quantum mechanics, whereas in Section 3, the relatively new Hamiltonian formulation of thermodynamics is reviewed, all of them in extended phase spaces and at the non-relativistic approximation.

### 2.1. Canonical Classical Mechanics

#### 2.1.1. Manifolds and Maps

**Figure 1:**
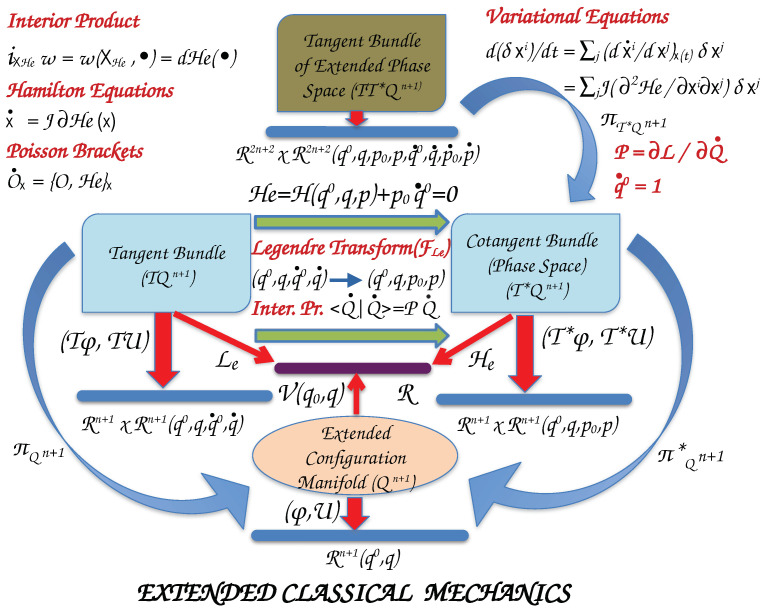
Manifolds and functions which determine the geometrical structures of a classical system with n+1 coordinates. $q^{0}$ denotes a parameter and, specifically, the time for time-dependent systems. $q=(q^{1},\ldots ,q^{n})$ are the *n* coordinates that define the configurations of the system and $p=(p_{1},\ldots ,p_{n})$ their canonical conjugate momenta. Details are given in the text.

We introduce the basic geometrical concepts of classical mechanics for time-dependent systems by elucidating the graphics of Figure 1. Starting from the bottom and moving upwards, we denote the set of configurations of the system with n–degrees of freedom (DOF) with the column vector (The symbol ${(}^{T})$ denotes the ***transpose*** of a matrix. Thus, a row vector becomes a column vector, and vice versa, a column vector is converted into a row vector.), $q={\left(q^{1},\ldots ,q^{n}\right)}^{T}$, and the parameter (time) with $q^{0}$. Capital letters designate the set of n+1 coordinates as a column vector, $Q={\left(q^{0},q\right)}^{T}$. These coordinates parametrize the ***Extended Configuration Manifold***, $Q^{n+1}$. Generally, this is a smooth (differentiable) ***nonlinear manifold***.

Manifolds generalize the geometrical objects of curves and surfaces in three-dimensional Euclidean space into N–dimensional (N>3) spaces. It took more than two centuries to develop the current mathematical definition of a manifold since it required the parallel development of various other branches of Mathematics, such as topology, geometry, and algebra [28].

The extended configuration space $Q^{n+1}$ can be described locally by a coordinate system (chart) *Q*, i.e., a homeomorphism

(1) $$\phi :U\subset Q^{n+1}\to \phi \left(U\right)\subset R^{n+1},$$

of an open set *U* of $Q^{n+1}$ onto an open set ϕ(U) of a Euclidean space $R^{n+1}$ of dimension n+1.

In Euclidean space, we understand the definition of a coordinate system in $R^{n+1}$ as

(2) $$q^{i}=\pi^{i}\circ \phi \;\;\mathrm{or}\;\;\phi \left(s\right)={\left(q^{0}\left(s\right),q^{1}\left(s\right),q^{2}\left(s\right),\ldots ,q^{n}\left(s\right)\right)}^{T}\in R^{n+1},$$

for every point s∈U, and $\pi^{i}$ are the ***canonical projections*** taken to be differentiable functions. ***Transition maps*** provide the transformation from one coordinate system to another for points that belong to the intersection of two different open subsets.

The ***tangent space*** of $Q^{n+1}$ at a point $s\in Q^{n+1}\left(T_{s}Q^{n+1}\right)$ is a vector space, and the union of all tangent spaces for all points *s* of $Q^{n+1}$ form the ***tangent bundle*** $\left(TQ^{n+1}\right)$ with the extended configuration space $Q^{n+1}$ to be the ***base space***

(3) $$TQ^{n+1}=\underset{s\in Q^{n+1}}{⋃}T_{s}Q^{n+1}.$$

The tangent bundle contains both the configuration manifold $Q^{n+1}$ and its tangent spaces $T_{s}Q^{n+1}$ called the ***fibers***, and it is a smooth manifold of dimension 2(n+1). Since $TQ^{n+1}$ is also a smooth manifold, a chart is defined by the diffeomorphism

(4) $$T\phi :TU\to \phi \left(U\right)\times R^{n+1}\subset R^{n+1}\times R^{n+1}.$$

Each coordinate system (ϕ,U) from the atlas of $Q^{n+1}$ induces a coordinate system (ϕ,TU) for $TQ^{n+1}$. This chart is said to be the bundle chart associated with (ϕ,U). The velocities $\left({\dot{q}}^{i}=dq^{i}/dt\right)$ live in this space.

The ***potential function***, V(Q), is a function on the configuration manifold to real numbers. The ***Lagrangian***, $L_{e}\left(Q,\dot{Q}\right)$, is a function on the tangent bundle to real numbers.

The ***dual space*** of $TQ^{n+1}$ (the set of all linear maps on tangent bundle to real numbers) is the ***cotangent bundle*** ($M=T^{*}Q^{n+1}$), also named ***phase space***. The phase space is a differentiable manifold of 2(n+1)–dimension for which the tangent bundle can also be defined with charts described by the generalized coordinates ($q^{i}$), the conjugate momenta (Notice that we use superscripts for coordinates and subscripts for momenta.) $\left(p_{j}\right)$, and their velocities, where

(5) $$p_{j}=\partial L_{e}/\partial {\dot{q}}^{j},\;\;j=0,\ldots ,n.$$

The ***Extended Hamiltonian***, $H_{e}\left(Q,P\right)$, is a function on the phase space to real numbers, $H_{e}:T^{*}Q^{n+1}\mapsto R$, obtained by a ***Legendre transformation*** ($F_{L_{e}}$) of the Lagrangian. We may consider that the Legendre transformation generates a differentiable map between the tangent and cotangent bundles of $Q^{n+1}$, $F_{L_{e}}:TQ^{n+1}\to T^{*}Q^{n+1}$. $\pi_{Q^{n+1}}$ and $\pi_{Q^{n+1}}^{*}$ are ***canonical projections*** to extended configuration manifold of tangent and cotangent bundles, respectively. The tangent bundle of phase space is denoted by $TT^{*}Q^{n+1}$ and $\pi_{T^{*}Q^{n+1}}$ is the canonical projection to phase space.

For a system of particles with n–DOF, we define the Lagrangian as the difference between kinetic energy K and potential energy

(6) $$L_{e}\left(Q,\dot{q}\right)=K-V=\frac{1}{2}\sum_{i,j=1}^{n}{\dot{q}}^{i}g_{ij}\left(q,m\right){\dot{q}}^{j}-V\left(Q\right),$$

with $g_{ij}\left(q,m\right)$ to be the ***kinetic metric tensor***, written as a function of coordinates *q* and the particle masses *m*. The metric is the ***non-degenerate, symmetric, covariant tensor rank-2*** that defines the kinetic energy. The momentum $p_{i}$ is the ***covector*** of the velocity ${\dot{q}}^{i}$,

(7) $$p_{i}=\frac{\partial K}{\partial {\dot{q}}^{i}}=\sum_{j=1}^{n}g_{ij}{\dot{q}}^{j},\;\;i=1,\ldots ,n,$$

which is a map from the tangent bundle ($TQ^{n}$) to the cotangent bundle ($T^{*}Q^{n}$). Obviously, for a diagonal unit metric, $g_{ij}=\delta_{ij}$, momenta are equal to velocities.

The velocities ${\dot{q}}^{i}$ can also be extracted from the momenta by the inverse tensor $g^{ij}$

(8) $${\dot{q}}^{i}=\sum_{j}g^{ij}p_{j},$$

where $\sum_{l}g_{il}g^{lj}=\delta_{i}^{j}$ (The components of Kronecker delta tensor, $\delta_{i}^{j}$, are equal to 1 for i=j and 0 for i≠j.).

Momenta may also be considered to be 1–forms which act on the tangent bundle to real numbers, $p:TQ^{n}\mapsto R$, by taking the ***interior product (contraction)*** of 1–forms with vector fields. Employing Dirac’s notation, we write

(9) $$<\dot{q}|\dot{q}>=\sum_{i=1}^{n}p_{i}\left({\dot{q}}^{i}\right)=\sum_{i=1}^{n}p_{i}{\dot{q}}^{i}=\sum_{i,j=1}^{n}{\dot{q}}^{i}g_{ij}{\dot{q}}^{j}=2K.$$

If $H\left(q^{0},q,p\right)=K\left(p\right)+V\left(Q\right)$ is the Hamiltonian of a system of particles with n–DOF, then the extended Hamiltonian, $\left(H_{e}\right)$, is defined with the Legendre transformation (Notice that in chemical thermodynamics the Legendre transformation is defined as the difference between the function and the sum of products of conjugate variables).

(10) $$H_{e}=P\dot{Q}-L_{e}=p_{0}{\dot{q}}^{0}+2K-K+V=p_{0}{\dot{q}}^{0}+H\left(q^{0},q,p\right).$$

The ***physical states*** are obtained by imposing the two constraints

(11) $$H_{e}=0,\;\;\;\;{\dot{q}}^{0}=1,$$

which result in

(12) $$p_{0}=-H\left(q^{0},q,p\right).$$

Hence, the time-extended system is a conservative system with Hamiltonian $H_{e}$.

#### 2.1.2. Equations of Motion

We collect the generalized coordinates $Q={\left(q^{0},q^{1},\ldots ,q^{n}\right)}^{T}$ and their conjugate momenta $P=(p_{0},p_{1},\ldots ,p_{n})$ to a single column vector $x={\left(Q,P^{T}\right)}^{T}$ of 2k–dimension, where we use k=n+1. ***Hamilton’s principle of stationary action*** leads to Hamilton’s equations of motion. Then, Hamilton’s equations with a Hamiltonian $H_{e}\left(x\right)$ are written in the form

(13) $$\dot{x}={\left(\dot{Q},{\dot{P}}^{T}\right)}^{T}={\left(\frac{\partial H_{e}\left(x\right)}{\partial P},-\frac{\partial H_{e}\left(x\right)}{\partial Q}\right)}^{T},$$

or

(14) $$\dot{x}\left(t\right)=J\partial H_{e}\left(x\left(t\right)\right),$$

where $\partial H_{e}\left(x\right)$ is the gradient of Hamiltonian function, and *J* the ***symplectic matrix***. *J* is the map on the tangent bundle of phase space *M*

(15) $$J:TM\to TM,\;\;\;\;J=\left(\begin{array}{cc} 0_{k} & I_{k}\\ -I_{k} & 0_{k} \end{array}\right).$$

$0_{k}$ and $I_{k}$ are the zero and unit k×k matrices, respectively. It is proved that *J* satisfies the relations,

(16) $$J^{-1}=-J=J^{T}\;and\;J^{2}=-I_{2k}.$$

Thus, the ***Hamiltonian vector field*** is

(17) $$X_{H_{e}}={\left(\frac{\partial H_{e}\left(x\right)}{\partial P},-\frac{\partial H_{e}\left(x\right)}{\partial Q}\right)}^{T},$$

or using the coordinate base in the tangent bundle of phase space $\left(Q,P,\frac{\partial }{\partial Q},\frac{\partial }{\partial P}\right)$, we write

(18) $$X_{H_{e}}=\left(\frac{\partial H_{e}\left(x\right)}{\partial P}\right)\frac{\partial }{\partial Q}-\left(\frac{\partial H_{e}\left(x\right)}{\partial Q}\right)\frac{\partial }{\partial P},$$

that lives in the tangent space of phase space.

In a more general approach, we can extract the Hamiltonian vector field as follows. In the cotangent space of extended coordinate manifold, let us denote with $\theta_{e}$ the 1–form acting on the phase space manifold $M=T^{*}Q^{k}$

(19) $$\theta_{e}:M\to T^{*}M:m\in M\to \theta_{em}\in T_{m}^{*}M,$$

and with α the 1–forms on the configuration manifold $Q^{k}$

(20) $$\alpha :Q\to M:r\in Q^{k}\to \alpha_{r}\in M.$$

Since α is a linear map from $Q^{k}$ to *M* and $\theta_{e}$ an 1–form on *M* we can ***pull-back*** $\theta_{e}$ to $Q^{k}$ to produce the 1–form $\alpha^{*}\theta_{e}$, which lives on the base manifold $Q^{k}$. Then, the ***canonical Poincaré* 1–*form*** satisfies the relation (***tautological* 1–*form***)

(21) $$\alpha^{*}\theta_{e}=\alpha \;\;\mathrm{for}\mathrm{all}\;\;\alpha .$$

Hence, we can expand $\theta_{e}$ as

(22) $$\theta_{e}=\sum_{i}p_{i}dq^{i}.$$

$\theta_{e}$ is invariant under coordinate transformations

(23) $$F^{i}=F^{i}\left(q^{0},q^{1},\ldots ,q^{n}\right),\;\;i=0,\ldots ,n,$$

which we assume to be invertible

(24) $$q^{j}=q^{j}\left(F^{0},\ldots ,F^{n}\right),\;\;j=0,\ldots ,n.$$

This is proved by arguing as follows. The velocities are given by

(25) $${\dot{q}}^{j}=\sum_{i}\left(\frac{\partial q^{j}}{\partial F^{i}}\right){\dot{F}}^{i}.$$

The new momenta are

(26) $$\begin{array}{ccc} P_{Fi} & = & \frac{\partial L_{e}}{\partial {\dot{F}}^{i}}=\sum_{j}\left(\frac{\partial L_{e}}{\partial {\dot{q}}^{j}}\right)\left(\frac{\partial {\dot{q}}^{j}}{\partial {\dot{F}}^{i}}\right)\\ & = & \sum_{j}p_{j}\left(\frac{\partial q^{j}}{\partial F^{i}}\right). \end{array}$$

Hence,

(27) $$\begin{array}{ccc} \theta_{e} & = & \sum_{i}p_{i}dq^{i}=\sum_{i}p_{i}\left(\sum_{j}\frac{\partial q^{i}}{\partial F^{j}}dF^{j}\right)\\ & = & \sum_{j}\left(\sum_{i}p_{i}\frac{\partial q^{i}}{\partial F^{j}}\right)dF^{j}=\sum_{j}P_{Fj}dF^{j}. \end{array}$$

The ***canonical Symplectic* 2–*form*** is extracted by taking the ***exterior derivative*** (Notice the negative sign in our formulation) of $\theta_{e}$

(28) $$\omega_{e}=-d\theta_{e}.$$

This is a ***non-degenerate, skew-symmetric, closed* 2–*form*** ($d\omega_{e}=-d\circ d\theta_{e}=0$). In local coordinates (q,p), $\omega_{e}$ is expressed by the ***wedge products***

(29) $$\omega_{e}=\sum_{i}dq^{i}\wedge dp_{i}.$$

If we introduce $dx=\left(dx^{1},\ldots ,dx^{2k}\right)=\left(dq^{0},\ldots ,dq^{n},dp_{0},\ldots ,dp_{n}\right)$, the symplectic 2–form (Equation (29)) is written

(30) $$\omega_{e}=\sum_{i=1}^{k}dx^{i}\wedge dx^{k+i}.$$

A pair $\left(M,\omega_{e}\right)$, i.e., the phase space with the symplectic 2–form, is said to be a ***symplectic manifold***. Those local coordinates which satisfy, $\omega_{e}=\sum_{i=1}^{k}dx^{i}\wedge dx^{k+i}$, are said to be ***canonical*** and ***symplectic***. In the following, we shall see that Hamiltonian mechanics and its geometrical properties can be formulated by $\omega_{e}$.

Let $\left(M,\omega_{e}\right)$ be a symplectic manifold of dimension 2k with $\omega_{e}$ a canonical symplectic 2–form. The Hamiltonian function $H_{e}$ is a smooth function on the phase space. The Hamiltonian vector field, $X_{H_{e}}$, (Equations (17) and (18)) is then defined via the relationship

(31) $$i_{X_{H_{e}}}\omega_{e}=\omega_{e}\left(X_{H_{e}},•\right)=dH_{e}\left(•\right).$$

$i_{X_{H_{e}}}\omega_{e}$ symbolizes ***interior product (contraction)*** and the triple $\left(M,\omega_{e},X_{H_{e}}\right)$ is a ***Hamiltonian system***. In particular, for the variable $q_{0}$ we extract the equation

(32) $${\dot{p}}_{0}=-\frac{\partial H(q^{0},q,p)}{\partial q^{0}}.$$

We can see that with this formulation of time-dependent systems and taking into account the constraints Equations (11) and (12), the trajectories are described at each time *t* in the physical phase space of the system of 2n–dimension. With $x_{q}={\left(q^{1},\ldots ,q^{n},p_{1},\ldots ,p_{n}\right)}^{T}$ Hamilton’s equations of motion with the time-dependent Hamiltonian are written

(33) $${\dot{x}}_{q}\left(t\right)=J\partial H\left(t,x_{q}\right).$$

For a smooth function O(x) on phase space, the ***Poisson bracket*** is defined as

(34) $$\left\{O,H_{e}\right\}=-\left\{H_{e},O\right\}=\omega_{e}\left(X_{O},X_{H_{e}}\right)=-dH_{e}\left(X_{O}\right).$$

Since

(35) $$\begin{array}{ccc} X_{O} & = & {\left(\frac{\partial O(x)}{\partial p},-\frac{\partial O(x)}{\partial q}\right)}^{T} \end{array}$$

(36) $$\begin{array}{ccc} X_{H_{e}} & = & {\left(\frac{\partial H_{e}\left(x\right)}{\partial p},-\frac{\partial H_{e}\left(x\right)}{\partial q}\right)}^{T}, \end{array}$$

or employing the coordinate base in the tangent space of phase space

(37) $$\begin{array}{ccc} X_{O} & = & \sum_{i=0}^{n}\left[\frac{\partial O}{\partial p_{i}}\frac{\partial }{\partial q_{i}}-\frac{\partial O}{\partial q_{i}}\frac{\partial }{\partial p_{i}}\right] \end{array}$$

(38) $$\begin{array}{ccc} X_{H_{e}} & = & \sum_{i=0}^{n}\left[\frac{\partial H_{e}}{\partial p_{i}}\frac{\partial }{\partial q_{i}}-\frac{\partial H_{e}}{\partial q_{i}}\frac{\partial }{\partial p_{i}}\right] \end{array}$$

we write the Poisson brackets in a coordinate system as

(39) $$\begin{array}{ccc} \left\{O,H_{e}\right\} & = & -dH_{e}\left(X_{O}\right)=-<dH_{e}|X_{O}>\\ & = & -\left(\frac{\partial H_{e}}{\partial q}dq+\frac{\partial H_{e}}{\partial p}dp\right)\left(\frac{\partial O}{\partial p}\frac{\partial }{\partial q}-\frac{\partial O}{\partial q}\frac{\partial }{\partial p}\right)\\ & = & \sum_{i=0}^{n}\left[\frac{\partial O}{\partial q^{i}}\frac{\partial H_{e}}{\partial p_{i}}-\frac{\partial O}{\partial p_{i}}\frac{\partial H_{e}}{\partial q^{i}}\right]. \end{array}$$

We have used

(40) $$dx_{i}\left(\frac{\partial }{\partial x^{j}}\right)=\delta_{i}^{j},\;\;\;\left(i,j\right)=1,\ldots ,2k.$$

where δ is the Kronecker’s delta tensor.

The ***Lie derivative*** of a dynamical quantity O(x(t)) with respect to the Hamiltonian vector field $X_{H_{e}}$ is defined as the directional derivative of O along the vector $X_{H_{e}}$

(41) $$L_{X_{H_{e}}}O=X_{H_{e}}\left(O\right)=dO\left(X_{H_{e}}\right)=\omega_{e}\left(X_{O},X_{H_{e}}\right)=\left\{O,H_{e}\right\}.$$

So,

(42) $$\begin{array}{ccc} \dot{O} & = & L_{X_{H_{e}}}O=X_{H_{e}}\left(O\right), \end{array}$$

(43) $$\begin{array}{ccc} \dot{O} & = & \{O,H_{e}\}. \end{array}$$

From the above equation, we infer that for conserved quantities, $\dot{O}=0$, the Poisson’s bracket commutes, i.e., $\{O,H_{e}\}=0$.

Applying the above equation to coordinates and conjugate momenta, (Q,P), we extract Hamilton’s equations

(44) $$\begin{array}{ccc} \dot{Q} & = & \left\{Q,H_{e}\right\}=\frac{\partial H_{e}}{\partial P} \end{array}$$

(45) $$\begin{array}{ccc} \dot{P} & = & \left\{P,H_{e}\right\}=-\frac{\partial H_{e}}{\partial Q}. \end{array}$$

Poisson brackets defined on a set of smooth functions F(M) on *M* satisfy the properties; For (f,g,h)∈F(M), then

- {f,g} is bilinear,
- {f,g}=−{g,f} antisymmetric,
- {f,f}=0, and
- {f,{g,h}}+{h,{f,g}}+{g,{h,f}}=0 (Jacobi identity).

#### 2.1.3. Integrable Hamiltonian Systems

In classical mechanics, a finite 2n–dimensional Hamiltonian system, $\left(M^{2n},\omega ,X_{H}\right)$ is ***completely integrable*** if it admits *n* independent constants of motion whose Poisson brackets are in involution, i.e., pairwise commute.

Let the integrals of motion are $F_{1}=H,\ldots ,F_{n}$ and are assigned to values $c=(c_{1},\ldots ,c_{n})$. Then, the corresponding level set, $F^{-1}\left(c\right)$ is a ***Lagrangian submanifold***.

In a more formal wording, for an even-dimensional phase space, *M*, the symplectic 2–form ω, which satisfies the condition (volume form)

$${\left(d\theta \right)}^{n}\neq 0,$$

the condition ω=0 determines the nD–Lagrangian submanifold $L_{p}^{n}\in M$.

For ***compact phase spaces***, the Lagrangian submanifolds have the structure of a n–torus, $T^{n}$. Moreover, in a neighborhood of every such invariant torus, one can find ***angle-action coordinates*** (ϕ,I),

(46) $$y^{i}=y^{i}\left(\phi ;I\right),\;\;\;\phi^{i},\in \left[0,2\pi \right),\;\;\;I_{j}=\frac{1}{2\pi }\oint p_{j}dq^{j},\;\;\;i,j=1,\ldots ,n.$$

In such a coordinate system, the Hamiltonian function depends only on $I_{j}$, i.e., H(I), and Hamilton’s equations give

(47) $$\begin{array}{ccc} {\dot{\phi }}^{i} & = & \frac{\partial H}{\partial I^{i}}=w_{i},\;\;\;\;\phi^{i}\left(t\right)=\phi_{0}^{i}+w_{i}t\\ {\dot{I}}^{i} & = & -\frac{\partial H}{\partial \phi^{i}}=0. \end{array}$$

$w_{i}$ are the normal frequencies, and the action variables are constants. The Poisson brackets of the action coordinates, *I*, pairwise commute, $\{I_{i},I_{j}\}=0$, and also satisfy $\left\{\phi^{i},I_{j}\right\}=\delta_{j}^{i}$.

#### 2.1.4. Complexification of Classical Hamilton’s Equations

Hamilton’s equations in classical mechanics can also be cast in a complex manifold by ***complexification*** of phase space, i.e., by introducing the complex transformation

$$\begin{array}{ccc} z_{l} & = & \frac{1}{\sqrt{2}}\left(q^{l}+𝚤p_{l}\right) \end{array}$$

(48) $$\begin{array}{ccc} z_{l}^{*} & = & \frac{1}{\sqrt{2}}\left(q^{l}-𝚤p_{l}\right) \end{array}$$

(49) $$\begin{array}{ccc} 𝚤 & = & \sqrt{-1}. \end{array}$$

Similarly, in Section 2.2.3 by ***realification*** of the quantum Hilbert space, we bring the Schrödinger equation into the form of Hamilton’s equations.

To make the complex transformation canonical, we define the ***complex variables***

(50) $$Q_{l}=z_{l},\;\;\;\;\;P_{l}=𝚤z_{l}^{*}.$$

The inverse transformation gives the real functions

(51) $$\begin{array}{ccc} q^{l} & = & \frac{1}{\sqrt{2}}\left(z_{l}+z_{l}^{*}\right)=\frac{1}{\sqrt{2}}\left(Q_{l}-𝚤P_{l}\right)\\ p_{l} & = & \frac{-𝚤}{\sqrt{2}}\left(z_{l}-z_{l}^{*}\right)=\frac{1}{\sqrt{2}}\left(P_{l}-𝚤Q_{l}\right). \end{array}$$

Because of the symmetry of Hamiltonians under the unitary group U(1)≅C, i.e., the Hamiltonian H should be invariant with a unitary transformation. We write the canonical Poincaré 1–form as

(52) $$\begin{array}{ccc} \theta_{c} & = & \sum_{l}\frac{1}{2}\left(p_{l}dq^{l}-q^{l}dp_{l}\right)=\sum_{l}\frac{𝚤}{2}\left(z_{l}^{*}dz_{l}-z_{l}dz_{l}^{*}\right) \end{array}$$

(53) $$\begin{array}{ccc} & = & \sum_{l}\frac{1}{2}\left(P_{l}dQ_{l}-Q_{l}dP_{l}\right). \end{array}$$

and thus, the canonical symplectic 2–form becomes

(54) $$\omega_{c}=-d\theta_{c}=\sum_{l}dq^{l}\wedge dp_{l}=\sum_{l}𝚤dz_{l}\wedge dz_{l}^{*}=\sum_{l}dQ_{l}\wedge dP_{l}.$$

For example, a harmonic oscillator in scaled normal coordinates and introducing these canonical complex coordinates results in

(55) $$\begin{array}{ccc} H & = & \frac{1}{2}w\left(q^{2}+p^{2}\right)\\ & = & \frac{1}{2}w\left[\frac{1}{2}\left(z^{2}+z^{*2}+2zz^{*}\right)-\frac{1}{2}\left(z^{2}+z^{*2}-2zz^{*}\right)\right] \end{array}$$

(56) $$\begin{array}{ccc} & = & wzz^{*}=-𝚤wQP=P\dot{Q}. \end{array}$$

Since the transformation to complex coordinates and conjugate momenta is symplectic, Hamilton’s equations are also written as

(57) $$\begin{array}{ccc} \dot{Q} & = & \frac{\partial H^{\prime }\left(Q,P\right)}{\partial P}=-𝚤wQ\\ \dot{P} & = & -\frac{\partial H^{\prime }\left(Q,P\right)}{\partial Q}=𝚤wP, \end{array}$$

where $H^{\prime }\left(Q,P\right)=H\left[q\left(Q,P\right),p\left(Q,P\right)\right]$ is the Hamiltonian in complex coordinates.

#### 2.1.5. Jacobi Fields and Variational Equations

Geodesic curves are obtained by searching for the critical points of the ***length functional***, i.e., by requiring

(58) $$\delta \int_{0}^{t_{max}}\left|\frac{dq(t)}{dt}\right|dt=\delta \int_{0}^{l_{max}}ds=0.$$

ds is the infinitesimal length of the curve given by the norm of the velocity vector.

The same critical points are obtained by varying the integrand ${\left|dq/dt\right|}^{2}$ instead

(59) $$\delta \int_{0}^{t_{max}}{\left|\frac{dq(t)}{dt}\right|}^{2}dt=\delta \int_{0}^{l_{max}}{\left(ds\right)}^{2}=0.$$

It is worth noting that the above integral depends on the parameter *t*. This integral is related to the ***action*** or the ***energy*** of a physical system. Indeed, Hamilton’s principle of stationary action results in Hamilton’s equations, the solutions of which are geodesics on the phase space manifold.

Let *C* be a trajectory in phase space with kinetic metric *g*

(60) $$\begin{array}{ccc} g(\dot{q},\dot{q}) & = & \sum_{i,j=1}^{n}g_{ij}dq^{i}⊗dq^{j}\left(\dot{q},\dot{q}\right)=\sum_{i,j=1}^{n}g_{ij}dq^{i}\left(\dot{q}\right)dq^{j}\left(\dot{q}\right)\\ & = & \sum_{i,j=1}^{n}g_{ij}{\dot{q}}^{i}{\dot{q}}^{j}=\sum_{i=1}^{n}{\dot{q}}^{i}\left(\sum_{j=1}^{n}g_{ij}{\dot{q}}^{j}\right)\\ & = & \sum_{i=1}^{n}p_{i}{\dot{q}}^{i}=\langle \dot{q}|\dot{q}\rangle . \end{array}$$

From the above equations, we deduce that the coefficients $g_{ij}$ result from the action of the metric on the base coordinates of the tangent space

(61) $$g_{ij}=g\left(\frac{\partial }{\partial q^{i}},\frac{\partial }{\partial q^{j}}\right).$$

The ***length of a trajectory*** starting from the point t=0 and finishing at the point $t=t_{max}$ is calculated as the line integral

(62) $$L\left(C\right)=\int_{0}^{t_{max}}\left|\dot{q}\right|dt=\int_{0}^{t_{max}}\sqrt{g\left(\dot{q},\dot{q}\right)}\;\;dt=\int_{0}^{t_{max}}\sqrt{\sum_{i=1}^{n}p_{i}{\dot{q}}^{i}}\;\;dt.$$

**Figure 2:**
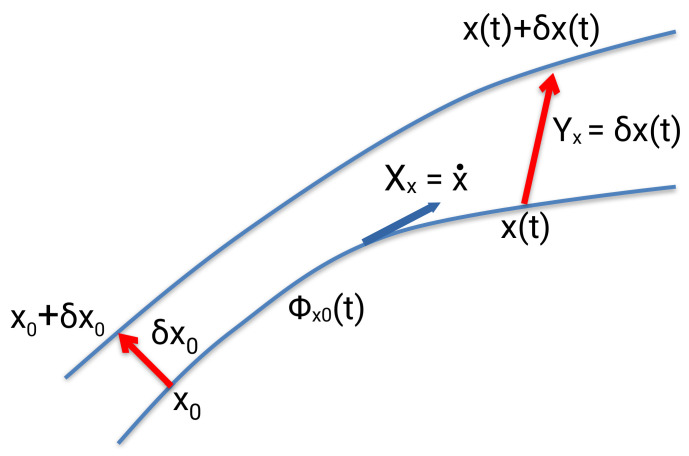
The description of the ***variation vector field***, $Y_{x}$, with respect to a reference trajectory with vector field $X_{x}$ and initial condition $x_{0}$. $\Phi_{x_{0}}\left(t\right)$ denotes the Hamiltonian flow.

In case we want to investigate the behavior of neighboring trajectories $C_{x+\delta x}$ to a reference one $C_{x}$ in phase space, we examine the time evolution of the ***variation vector*** $Y_{x}\left(t\right)=\delta x\left(t\right)$, (Figure 2). The time derivative of the vector field $Y_{x}\left(t\right)$ with respect to the vector field $X_{x}$ (Lie derivative) is equal to

(63) $$\begin{array}{ccc} {\dot{Y}}_{x}^{i}=\frac{d(\delta x^{i})}{dt} & = & \left({\dot{x}}^{i}+\frac{d(\delta x^{i})}{dt}\right)-{\dot{x}}^{i}=X_{x+\delta x}^{i}-X_{x}^{i}\\ & = & \sum_{j}\left(\frac{\partial {\dot{x}}^{i}}{\partial x^{j}}\right){|}_{x(t)}\delta x^{j}\left(t\right)+h.o.t.\\ & = & \sum_{j}J\left(\frac{\partial^{2}H_{e}}{\partial x^{i}\partial x^{j}}\right){|}_{x(t)}\delta x^{j}\left(t\right)+h.o.t.. \end{array}$$

*J* is the symplectic matrix (Equation (15)). The higher order term (h.o.t.) is a function of the displacement δx at time *t*, which contains all the terms in the Taylor expansion larger than the first order. The derivatives are computed at the reference trajectory with initial conditions $x_{0}$. $Y_{x}=\delta x$ is a ***Jacobi field*** and the equations

(64) $$\frac{dY_{x}^{i}}{dt}=\sum_{j}\left(\frac{\partial X^{i}}{\partial x^{j}}\right){|}_{x(t)}Y^{j},$$

the ***variational equations.***

The ***fundamental matrix*** $Z(t,t_{0})$ satisfies the variational equations with initial condition $Z(t_{0},t_{0})=I$. It is a symplectic matrix, and therefore, the following equations are valid [3]

(65) $$\det\left(Z\right)=1,\;\;\;\;Z^{T}JZ=J.$$

It is proved that if σ is an eigenvalue of a real symplectic matrix, so are $\sigma^{-1},\;\;\sigma^{*}$ and ${\left(\sigma^{*}\right)}^{-1}$. $\sigma^{*}$ are complex conjugate numbers. Also, for every constant of motion, two eigenvalues of the fundamental matrix are equal to one. For proofs and applications see references [4,5,29,30].

### 2.2. Geometrical Quantum Mechanics

#### 2.2.1. Manifolds and Maps

**Figure 3:**
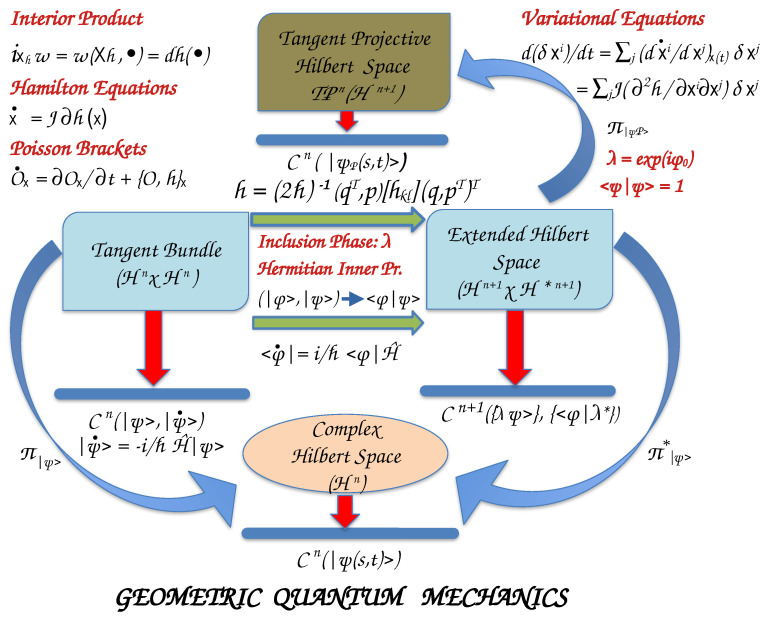
Manifolds and functions which determine the geometrical structures of a quantum system. The states of the quantum system are the elements of a n–dimensional complex vector space (Hilbert space $H^{n}≅C^{n})$ that includes the vectors |ψ>, their complex conjugate <ψ|, and linear transformations $|\dot{\psi }>$. Hermitian inner products, <ϕ|ψ>, are employed for Hilbert spaces. The tangent bundle $\left(H^{n}\times H^{n}\right)$ is mapped to the Extended Hilbert Space $\left(H^{n+1}\times H^{n+1}\right)$ by the inclusion of a complex phase λ, the elements of the unitary group U(1), that produces the rays {|ψ>}:={λ|ψ>}. The canonical projection, $\pi_{|\psi_{P}>}$, projects the rays in $H^{n+1}$ onto the Projective Hilbert Space $P^{n}\left(H^{n+1}\right)$, the space where the physical states of the system live. Details are given in the text.

Moving to the quantum world, the states of a system are described by complex vectors, |ψ(s,t)>, with *s* to be the configuration coordinates and *t* the time instead of the real coordinates and momenta (or velocities) in classical mechanics. The square of the norm of state vectors is interpreted as a probability density, with ${\left||\psi \left(s,t\right)>\right|}^{2}ds$ to be the probability of finding the system in the coordinate intervals [s,s+ds] at time *t*. The observable quantities, which are real-valued functions on phase space in classical mechanics, are replaced by operators that designate linear transformations on the complex state vector space, named Hilbert space of dimension *n* ($H^{n}≅C^{n}$) (Figure 3). Infinite-dimensional Hilbert spaces for quantum systems also exist.

The time evolution of the quantum states is given by Schrödinger’s equation

(66) $$\frac{\partial }{\partial t}\left|\psi >=\right|\dot{\psi }>=-\frac{𝚤}{\hbar }\hat{H}\left|\psi >=X_{\hat{H}}\right|\psi >,$$

where $𝚤=\sqrt{-1}$, *ℏ* the reduced Heisenberg constant, $\hat{H}$ is the ***Hamiltonian operator*** of the system that corresponds to its energy, and $X_{\hat{H}}=-\frac{𝚤}{\hbar }\hat{H}$ is the ***Hamiltonian–Schrödinger vector field***. To any observable O, we assign the operator $\hat{O}$ and the ***Schrödinger vector field*** $X_{\hat{O}}=-\frac{𝚤}{\hbar }\hat{O}$. Hence, we may consider the vectors |ψ> as ***vector fields***, and thus, the Hilbert space $H^{n}$ to be also the tangent space $T_{|\psi >}H^{n}$ at the state $|\psi >\in H^{n}$.

The cotangent space of the Hilbert space, $T_{|\psi >}^{*}H^{n}$, at state |ψ> is $H^{*n}$, with covectors to be the complex conjugate functionals $<\phi |\;\in \;T_{|\psi >}^{*}H^{n}$, and <ϕ|ψ>↦C.

<ϕ|ψ> (We use Dirac’s (bra, ket) notation.) denotes the ***Hermitian inner product***, ($<\phi |\psi >=<\psi |\phi {>}^{*}$).

Separating the real and imaginary parts of the Hermitian inner product, we write

(67) $$\begin{array}{ccc} <\phi |\psi > & = & <\phi_{r}-𝚤\phi_{i}|\psi_{r}+𝚤\psi_{i}>\\ & = & \left[\left(\phi_{r},\psi_{r}\right)+\left(\phi_{i},\psi_{i}\right)\right]+𝚤\left[\left(\phi_{r},\psi_{i}\right)-\left(\phi_{i},\psi_{r}\right)\right]\\ & = & \sum_{k=1}^{n}\left(\phi_{r}^{k}\psi_{r}^{k}+\phi_{i}^{k}\psi_{i}^{k}\right)+𝚤\sum_{k=1}^{n}\left(\phi_{r}^{k}\psi_{i}^{k}-\phi_{i}^{k}\psi_{r}^{k}\right)\\ & = & G(|\phi >,|\psi >)+\Omega (|\phi >,|\psi >). \end{array}$$

$\left(\phi_{r}^{k},\psi_{r}^{k}\right)$ are the real and $\left(\phi_{i}^{k},\psi_{i}^{k}\right)$ the imaginary components, respectively, of the *k*th component of the complex vectors. *G* is a Riemannian metric, i.e., it is real, positive definite, and strongly non-degenerate

(68) $$G\left(|\phi >,|\psi >\right)=\left(\sum_{k}\left(\phi_{r}^{k}\psi_{r}^{k}+\phi_{i}^{k}\psi_{i}^{k}\right)\right).$$

Ω is a symplectic (antisymmetric) closed 2–form, and strongly non-degenerate

(69) $$\Omega \left(|\phi >,|\psi >\right)=\left(\sum_{k}\left(\phi_{r}^{k}\psi_{i}^{k}-\phi_{i}^{k}\psi_{r}^{k}\right)\right).$$

Both G,Ω act on the tangent bundle $TH^{n}$.

Since the physical interpretation of a quantum state is probabilistic, i.e., for a pure state |ψ> (the states of an isolated system), the following normalization condition should be satisfied

(70) $${<\psi |\psi >=||\psi >|}^{2}=1.$$

The observables, or measurable quantities of the system, O, are represented by ***self-adjoint linear operators***, $\hat{O}={\hat{O}}^{†}$, on $H^{n}$, and are thus vector fields. The ***expectation value*** of an observable with operator $\hat{O}$ at the state |ψ> is the ***real-valued function***

(71) $$O=\frac{<\psi \left(s,t\right)|\hat{O}|\psi \left(s,t\right)>}{<\psi |\psi >}=<\psi \left(s,t\right)|\hat{O}|\psi \left(s,t\right)>=\frac{1}{2\hbar }G(|\psi >,\hat{O}\left|\psi >\right).$$

In particular, for the Hamiltonian operator of the system, we write

(72) $$H=\frac{<\psi |\hat{H}|\psi >}{<\psi |\psi >}=<\psi |\hat{H}|\psi >=\frac{1}{2\hbar }G(|\psi >,\hat{H}\left|\psi >\right).$$

#### 2.2.2. Projective Hilbert Space

For any nonzero factor λ∈U(1)≅C, such as $\lambda =\exp\left(𝚤\phi_{0}\right)$, λ|ψ> yields the same expectation value for the observable O as does |ψ>. λ are the elements of the unitary Lie group, U(1). Thus, the inclusion of these phases to the initial Hilbert space results in the ***extended Hilbert space*** of (n+1)–dimension, $H^{n+1}$, which is isomorphic to $C^{n+1}$ with a Hermitian inner product.

The set of vectors obtained by multiplying the state |ψ> with λ consists of a 1–dimensional subspace of $H^{n+1}$, called ***ray***, which we symbolize as {|ψ>}≡{λ|ψ>}. A ray is an ***equivalence class of vectors*** in $H^{n+1}$: two vectors are equivalent if and only if one is a nonzero complex scalar multiple of the other. Also, adopting normalized vectors (Equation (70)), the ***physical quantum states*** are elements of the complex ***Projective Hilbert space***, $P^{n}\left(H^{n+1}\right)$ of n–dimension obtained by the canonical projection map $\pi_{|\psi_{P}>}$ of the extended Hilbert space $H^{n+1}$.

Hence, a quantum system may be described with what mathematicians call ***principal bundle***

(73) $$U\left(1\right)≅C↪\left(H^{n}-\left\{0\right\}\right)\overset{\pi_{|\psi_{P}>}}{⟶}P^{n}\left(H^{n+1}\right),$$

with state vectors the rays {|ψ>} in $H^{n+1}$, and physical states $|\psi_{P}>$ in $P^{n}\left(H^{n+1}\right)$ obtained by the projection map $\pi_{|\psi_{P}>}$. The inverse projection is

(74) $$\pi_{|\psi_{P}>}^{-1}=\left\{\frac{e^{𝚤\phi_{0}}|\psi >}{<\psi |\psi >}\right\}=\left\{e^{𝚤\phi_{0}}|\psi >\right\}.$$

To recapitulate, the inclusion of the unitary group U(1) extends the n–dimensional Hilbert space $H^{n}$ to the $H^{n+1}$ extended Hilbert space. For normalized state vectors, the extended Hilbert space is mapped to the unit sphere $S^{2n+1}$ of dimension (2n+1) embedded in the real Euclidean $R^{2n+2}$ space. Finally, by the projection $\pi_{|\psi_{P}>}$ we produce the Projective Hilbert space isomorphic to the complex space $C^{n}$ or to real of 2n–dimensional space, $R^{2n}$,

(75) $$U\left(1\right)↪S^{2n+1}\overset{\pi_{|\psi_{P}>}}{⟶}P^{n}\left(H^{n+1}\right)≅C^{n},\;\;\;\;\;S^{2n+1}:\left\{|\psi >\right\}\in H^{n+1},\;\;<\psi |\psi >=1.$$

The ***tangent space of the projective space*** at point $|\psi_{P}>$, $T_{|\psi_{P}>}\left(P^{n}\left(H^{n+1}\right)\right)$, is isomorphic to the kernel of the ray, ${\left\{|\psi >\right\}}^{⊥}$, i.e.,

(76) $${\left\{|\psi >\right\}}^{⊥}=\{|\phi >\in H^{n+1};\;\;<\phi \left|\psi >=0\right\},$$

and the push-forward of the projection map, $\pi_{*}^{⊥}\left(\left\{|\psi >\right\}\right)$, is a ***complex conjugate*** linear isomorphism onto $T_{|\psi_{P}>}\left(P^{n}\left(H^{n+1}\right)\right)$

(77) $$\pi_{*}^{⊥}:{\left\{|\psi >\right\}}^{⊥}\to T_{|\psi_{P}>}\left(P^{n}\left(H^{n+1}\right)\right).$$

An illustrative figure is shown in the article of Dorje C. Brody and Lane P. Hughston [7].

For a normalized ray and its kernel, one can argue that [6,7,8]

(78) $$<\pi_{*}^{⊥}\phi_{1}|\pi_{*}^{⊥}\phi_{2}{>}_{T_{|\psi_{P}>}\left(P^{n}\left(H^{n+1}\right)\right)}=<\phi_{1}|\phi_{2}{>}_{H^{n+1}},\;\;\;\;(|\phi_{1}>,\left|\phi_{2}>\right)\in {\left\{|\psi >\right\}}^{⊥},$$

which gives a well-defined Hermitian inner product on $T_{|\psi_{P}>}\left(P^{n}\left(H^{n+1}\right)\right)$. Moreover, we deduce that the equation

(79) $$\omega_{|\psi_{P}>}(\pi_{*}^{⊥}|\phi_{1}>,\pi_{*}^{⊥}|\phi_{2}>)=ℑ<\phi_{1}|\phi_{2}>=\Omega_{\{|\psi >\}⊥}\left(\right|\phi_{1}>,\left|\phi_{2}>\right),$$

provides a strong symplectic form on $P^{n}\left(H^{n+1}\right)$, and the equation

(80) $$g_{|\psi_{P}>}(\pi_{*}^{⊥}|\phi_{1}>,\pi_{*}^{⊥}|\phi_{2}>)=ℜ<\phi_{1}|\phi_{2}>=G_{\{|\psi >\}⊥}\left(\right|\phi_{1}>,\left|\phi_{2}>\right),$$

defines a strong Riemannian metric on $P^{n}\left(H^{n+1}\right)$ called the ***Fubini–Study metric***. *ℜ* and *ℑ* are the real and imaginary parts of the Hermitian inner product, respectively, in the extended complex Hilbert space $H^{n+1}$. Both $\omega_{|\psi_{P}>}$ and $g_{|\psi_{P}>}$ are invariant under all transformations U(1), for all unitary operators $\hat{U}$ on $H^{n+1}$.

#### 2.2.3. Realification of Hilbert Space and Kähler Manifolds

Expanding the vectors of Hilbert space in a n–dimensional basis, as well as its dual basis, we write the n–coefficients $\psi^{k}$ with the real $\left(\psi_{r}^{k}\right)$ and imaginary $\left(\psi_{i}^{k}\right)$ components. Similarly, the covectors are written as $\left(\psi_{kr}\right)$ for the real and $\left(\psi_{ki}\right)$ for the imaginary components. Thus, working with the real vectors ${\left(\psi_{r},\psi_{i}\right)}^{T}$ we can describe quantum states in a real vector space $H_{r}^{2n}≅R^{n}\times R^{n}\equiv R^{2n}$.

An ***almost complex structure*** is introduced in $H_{r}^{2n}$ by replacing the imaginary number $𝚤=\sqrt{-1}$ with the symplectic matrix −J, Equation (15). We also derive

(81) $$\begin{array}{ccc} G(|\phi >,|\psi >) & = & G(J|\phi >,J|\psi >) \end{array}$$

(82) $$\begin{array}{ccc} \Omega (|\phi >,|\psi >) & = & \Omega (J|\phi >,J|\psi >) \end{array}$$

(83) $$\begin{array}{ccc} G(|\phi >,|\psi >) & = & \Omega (J|\phi >,|\psi >)=-\Omega (|\phi >,J|\psi >) \end{array}$$

(84) $$\begin{array}{ccc} \Omega (|\phi >,|\psi >) & = & G(|\phi >,J|\psi >). \end{array}$$

The triple (J,G,Ω) attributes a ***Kähler structure***, and thus, $H_{r}^{2n}$ is a ***Kähler manifold*** [2].

By realification of the Hilbert space, we may express the Hamiltonian–Schrödinger vector field as

(85) $$X_{\hat{H}}=\frac{J}{\hbar }\hat{H}.$$

Also, for an observable, O, the corresponding Schrödinger vector field is

(86) $$X_{\hat{O}}\left|\psi >=\frac{J}{\hbar }\hat{O}\right|\psi >.$$

For a time-independent Hamiltonian, $\hat{H}$, we solve the Schrödinger equation by employing the unitary propagator $\hat{U}\left(t\right)=\exp\left(-𝚤\hat{H}t/\hbar \right)$, and write the Hamiltonian flow as

(87) $$|\psi \left(s,t\right)>=\hat{U}\left(t\right)\left|\psi_{0}>=\exp\left[-\frac{𝚤}{\hbar }t\hat{H}\right]\right|\psi_{0}>.$$

$\hat{U}$ generates a one-parameter group of transformations on $H_{r}^{2n}$, which preserve the metric *G* and the symplectic 2–form Ω.

The triple $\left(H_{r}^{2n},\Omega ,X_{\hat{H}}\right)$ is a Hamiltonian system with Hamiltonian function the ***Expectation value*** of $\hat{H}$ at the state |ψ>

(88) $$H\left(t\right)=\frac{<\psi \left(s,t\right)|\hat{H}|\psi \left(s,t\right)>}{<\psi |\psi >}=\frac{1}{2\hbar }\;G(|\psi >,\hat{H}\left|\psi >\right).$$

We can prove (see Appendix A.1) that for an observable $\hat{O}$ and normalized states <ψ|ψ>=1, the differential 1–form of the expectation value of $\hat{O}$ is the interior product of the symplectic 2–form $\Omega (X_{\hat{O}},|\phi >)$ with the Schrödinger vector field $X_{\hat{O}}$ (Equation (86))

(89) $$dO\left(|\phi >\right)=i_{X_{\hat{O}}}\Omega \left(|\phi >\right),$$

where $|\phi >\;\in \;{\left\{|\psi >\right\}}^{⊥}$. The above equation is in accordance with Equation (31) of classical mechanics. Hence, Hermitian operators $\hat{O}$ give rise to quadratic real-valued functions O(|ψ>). The vector field $X_{\hat{O}}$ generated by the expectation value function of the operator $\hat{O}$ is a Schrödinger (Hamiltonian) field.

If $\left(O_{1},O_{2}\right)$ are the expectation value functions of two observables, the Poisson bracket (Equation (39)) is defined by the equation (see Appendix A.2)

(90) $$\left\{O_{1},O_{2}\right\}=\Omega \left(X_{{\hat{O}}_{1}},X_{{\hat{O}}_{2}}\right)=<\psi \left|-\frac{𝚤}{\hbar }\left[{\hat{O}}_{1},{\hat{O}}_{2}\right]\right|\psi >.$$

$\left[{\hat{O}}_{1},{\hat{O}}_{2}\right]$ is the commutator of the two operators $\left({\hat{O}}_{1},{\hat{O}}_{2}\right)$.

The uncertainty (***dispersion***) of a quantum observable $\hat{O}$ in a normalized state $|\psi >\in H^{n}$ is defined as [8,31]

(91) $${\left(\Delta O\right)}^{2}\left(|\psi >\right)=<\psi |{\hat{O}}^{2}\left|\psi >-(<\psi \right|\hat{O}{\left|\psi >\right)}^{2}=<\psi \left|{\hat{O}}^{2}\right|\psi >-\;O^{2}.$$

If |ψ> is an eigenvalue of the observable $\hat{O}$, then, $\left(\Delta \hat{O}\right)\left(|\psi >\right)=0$.

For two quantum observables $\left({\hat{O}}_{1},{\hat{O}}_{2}\right)\;\;\mathrm{on}\;\;H^{n}$ the product of the uncertainties of the expectation value functions $\left(O_{1},O_{2}\right)\mapsto R,\;\;O_{1}=<\psi |{\hat{O}}_{1}|\psi >,\;O_{2}=<\psi \left|{\hat{O}}_{2}\right|\psi >$ is written as

(92) $$\begin{array}{ccc} {\left(\Delta O_{1}\right)}^{2}{\left(\Delta O_{2}\right)}^{2} & = & \left[<\psi |{\hat{O}}_{1}^{2}\left|\psi >-(<\psi \right|{\hat{O}}_{1}{\left|\psi >\right)}^{2}\right]\left[<\psi |{\hat{O}}_{2}^{2}\left|\psi >-(<\psi \right|{\hat{O}}_{2}{\left|\psi >\right)}^{2}\right]\\ & = & \left[<\psi |{\hat{O}}_{1}^{2}|\psi >-\;O_{1}^{2}\right]\left[<\psi |{\hat{O}}_{2}^{2}|\psi >-\;O_{2}^{2}\right], \end{array}$$

and the ***covariance or correlation function*** is expressed as

(93) $$\begin{array}{ccc} C(O_{1},O_{2}) & = & \frac{1}{2}\left(<\psi |({\hat{O}}_{1}{\hat{O}}_{2}+{\hat{O}}_{2}{\hat{O}}_{1})|\psi >\right)-O_{1}O_{2}\\ & = & \frac{1}{2}\left(<\psi |{\left[{\hat{O}}_{1},{\hat{O}}_{2}\right]}_{+}|\psi >\right)-O_{1}O_{2}\\ & = & \frac{\hbar }{2}G\left(X_{{\hat{O}}_{1}},X_{{\hat{O}}_{2}}\right)-O_{1}O_{2}, \end{array}$$

where the anticommutator is denoted by ${\left[{\hat{O}}_{1},{\hat{O}}_{2}\right]}_{+}=\left({\hat{O}}_{1}{\hat{O}}_{2}+{\hat{O}}_{2}{\hat{O}}_{1}\right)$. It is proved that

(94) $$<\psi |{\left[{\hat{O}}_{1},{\hat{O}}_{2}\right]}_{+})|\psi >=\hbar G\left(X_{{\hat{O}}_{1}},X_{{\hat{O}}_{2}}\right).$$

The ***Schrödinger vector fields*** are expressed as usually

(95) $$\begin{array}{ccc} X_{{\hat{O}}_{1}} & = & \frac{\partial O_{1}}{\partial p}\frac{\partial }{\partial q}-\frac{\partial O_{1}}{\partial q}\frac{\partial }{\partial p}\\ X_{{\hat{O}}_{2}} & = & \frac{\partial O_{2}}{\partial p}\frac{\partial }{\partial q}-\frac{\partial O_{2}}{\partial q}\frac{\partial }{\partial p}. \end{array}$$

If we use Equation (90), then we can express the commutator

(96) $$<\psi |\left[{\hat{O}}_{1},{\hat{O}}_{2}\right]|\psi >=𝚤\hbar \Omega \left(X_{{\hat{O}}_{1}},X_{{\hat{O}}_{2}}\right).$$

***Schwartz inequality*** implies

(97) $$\begin{array}{ccc} {\left(\Delta O_{1}\right)}^{2}{\left(\Delta O_{2}\right)}^{2} & \geq & {\left[<\psi |\left({\hat{O}}_{1}|\psi >-O_{1}\right)\left(<\psi \right|{\hat{O}}_{2}\left|\psi >-O_{2}\right)\right]}^{2}\\ & = & {\left|\frac{1}{2}<\psi \left|\left[\left({\hat{O}}_{1}-O_{1}\right),\left({\hat{O}}_{2}-O_{2}\right)\right]\right|\psi >\right|}^{2}+\\ & & {\left|\frac{1}{2}<\psi |[\left({\hat{O}}_{1}-O_{1}\right),{\left({\hat{O}}_{2}-O_{2}\right)}_{+}|\psi >\right|}^{2}. \end{array}$$

Since

$$<\psi |\left[\left({\hat{O}}_{1}-O_{1}\right),\left({\hat{O}}_{2}-O_{2}\right)\right]\left|\psi >=<\psi \right|\left[{\hat{O}}_{1},{\hat{O}}_{2}\right]|\psi >=𝚤\hbar \Omega \left(X_{{\hat{O}}_{1}},X_{{\hat{O}}_{2}}\right),$$

and

(98) $$\begin{array}{ccc} <\psi |\left[{\hat{O}}_{1}-O_{1}\right),{\hat{O}}_{2}-O_{2}{]}_{+}|\psi > & = & <\psi |{\left[{\hat{O}}_{1},{\hat{O}}_{2}\right]}_{+}|\psi >-2O_{1}O_{2}\\ & = & \hbar G\left(X_{{\hat{O}}_{1}},X_{{\hat{O}}_{2}}\right)-2O_{1}O_{2}, \end{array}$$

the ***Robertson-Schrödinger uncertainty relation*** is written as (Notice that to be consistent with the literature in discussing the covariance of two observables, we introduce the factor 1/2 in the commutator and anticommutator).

(99) $$\begin{array}{ccc} {\left(\Delta O_{1}\right)}^{2}{\left(\Delta O_{2}\right)}^{2} & \geq & {\left[C(O_{1},O_{2})\right]}^{2}+{\left|\frac{1}{2𝚤}<\psi |\left[{\hat{O}}_{1},{\hat{O}}_{2}\right]\left|\psi >\right]\right|}^{2}\\ & = & {\left[\frac{\hbar }{2}G\left(X_{{\hat{O}}_{1}},X_{{\hat{O}}_{2}}\right)-O_{1}O_{2}\right]}^{2} \end{array}$$

(100) $$\begin{array}{ccc} & + & {\left[\frac{\hbar }{2}\Omega \left(X_{{\hat{O}}_{1}},X_{{\hat{O}}_{2}}\right)\right]}^{2}. \end{array}$$

#### 2.2.4. Equations of Motion

We have seen that in the Extended Hilbert space, the vector states are the rays {|ψ>}, which for simplicity we symbolize as |ψ>. The projection of the Extended Hilbert space on the n–dimensional tangent Projective Hilbert phase makes the solutions of the time-dependent Schrödinger equation to be equivalent to the solutions of Hamilton’s equations in the Projective Hilbert space, with the expectation value of the Hamiltonian operator playing the role of Hamiltonian function. Furthermore, the phase space is a Kähler manifold, both in the extended Hilbert space as well as in the Projective Hilbert space.

If we use as basis set the coordinate basis |s> the representation of the state |ψ> is

(101) $$|\psi >=\int_{-\infty }^{\infty }\psi \left(s\right)|s>ds.$$

The coefficient ψ(s) is called ***wavefunction*** and it is a complex function, $\psi \left(s\right)\;\in C^{n}$, which we assume normalized to one

(102) $$<\psi |\psi >=<\psi |\left(\int_{-\infty }^{\infty }\left|s><s\right|ds\right)|\psi >=\int_{-\infty }^{\infty }\psi {\left(s\right)}^{*}\psi \left(s\right)ds=\int_{-\infty }^{\infty }{\left|\psi \left(s\right)\right|}^{2}ds=1.$$

With an orthonormal coordinate basis, the ***completeness relation*** is written as

(103) $$\int_{-\infty }^{\infty }\left|s><s\right|ds=\hat{I}.$$

In the following, we adopt the coordinate representation of the state vectors using wavefunctions in the ***Schrödinger picture***, ψ(s,t). We expand |ψ(s,t)> of a dynamical system in an arbitrary orthonormal basis set, $|\chi_{k}\left(s\right)>,\;\;k=1,\ldots ,n$

(104) $$|\psi \left(s,t\right)>=\sum_{k=1}^{n}c_{k}\left(t\right)|\chi_{k}\left(s\right)>.$$

In this expansion, the basis functions $|\chi_{k}>$ are time-independent, whereas the coefficients $c_{k}$ depend on time. |ψ> are solutions of the Schrödinger equation evolving in the extended Hilbert space

(105) $$\begin{array}{c} 𝚤\hbar \frac{\partial |\psi (s,t)>}{\partial t}=\hat{H}|\psi \left(s,t\right)>, \end{array}$$

and their complex conjugate solutions of the equation

(106) $$\begin{array}{c} -𝚤\hbar \frac{\partial <\psi (s,t)|}{\partial t}=<\psi \left(s,t\right)|\hat{H}. \end{array}$$

We assume |ψ> to be normalized, <ψ(s,t)|ψ(s,t)>=1, at any time. Then, by substituting Equation (104) in the expectation value of the Hamiltonian, H, we obtain

(107) $$\begin{array}{ccc} H=<\psi |\hat{H}|\psi > & = & \int_{-\infty }^{\infty }\psi^{*}\hat{H}\psi ds\\ & = & \sum_{k}\sum_{l}c_{k}^{*}c_{l}\int_{-\infty }^{\infty }\chi_{k}^{*}\hat{H}\chi_{l}ds. \end{array}$$

The Schrödinger equation in the extended Hilbert space is mapped into Hamilton’s equations in the Projective Hilbert space $P^{n}\left(H^{n+1}\right)$ with Hamiltonian function the expectation value of the Hamiltonian operator $\hat{H}$. For simplicity, we again denote the quantum states $|\psi_{P}>$ in the Projective Hilbert space with |ψ>. Then, we differentiate Equation (107) with respect to $c_{k}^{*}$

(108) $$\begin{array}{ccc} \frac{\partial H}{\partial c_{k}^{*}} & = & \sum_{l}c_{l}<\chi_{k}\left|\hat{H}\right|\chi_{l}>\\ & = & <\chi_{k}\left|\hat{H}\right|\psi >\\ & = & 𝚤\hbar \frac{dc_{k}}{dt},\;\;k=1,\ldots ,n. \end{array}$$

Similarly, we take

(109) $$\begin{array}{ccc} \frac{\partial H}{\partial c_{l}} & = & \sum_{k}c_{k}^{*}<\chi_{k}\left|\hat{H}\right|\chi_{l}>\\ & = & <\psi \left|\hat{H}\right|\chi_{l}>\\ & = & -𝚤\hbar \frac{dc_{l}^{*}}{dt},\;\;l=1,\ldots ,n. \end{array}$$

We define the ***complex variables*** ($Q_{k},P_{k}$) by introducing the real functions $q^{k}\left(t\right)$ and $p_{k}\left(t\right)$ to correspond to real and imaginary parts of the complex variables, respectively,

(110) $$\begin{array}{ccc} Q_{k}\left(t\right) & = & c_{k}\left(t\right)=\frac{1}{\sqrt{2}}\left[q^{k}\left(t\right)+𝚤p_{k}\left(t\right)\right]\\ P_{k}\left(t\right) & = & 𝚤\hbar c_{k}^{*}\left(t\right)=\frac{𝚤\hbar }{\sqrt{2}}\left[q^{k}\left(t\right)-𝚤p_{k}\left(t\right)\right]=\frac{\hbar }{\sqrt{2}}\left[p_{k}\left(t\right)+𝚤q^{k}\left(t\right)\right]. \end{array}$$

Equations (108) and (109) are the quantum equivalent of Hamilton’s equations of motion

(111) $$\begin{array}{ccc} \dot{Q_{k}} & = & \frac{\partial H(Q,P)}{\partial P_{k}}\\ \dot{P_{k}} & = & -\frac{\partial H(Q,P)}{\partial Q_{k}} \end{array}$$

(112) $$\begin{array}{ccc} H(Q,P) & = & -\frac{𝚤}{\hbar }\left[\sum_{k}\sum_{l}P_{k}\left(\int_{-\infty }^{\infty }\chi_{k}^{*}\hat{H}\chi_{l}dx\right)Q_{l}\right]\\ & = & -\frac{𝚤}{\hbar }\sum_{k}\sum_{l}P_{k}h_{kl}Q_{l}=\left(-𝚤\sum_{k}\sum_{l}P_{k}w_{kl}Q_{l}\right)\\ & = & -\frac{𝚤}{\hbar }\sum_{k}P_{k}{\dot{Q}}_{k}. \end{array}$$

Hence,

(113) $$H\left(Q,P\right)=-\frac{𝚤}{\hbar }P\dot{Q}.$$

$w_{kl}=h_{kl}/\hbar$ are the ***transition frequencies***. It is also worth noting that the quantum Hamiltonian is similar to that of a harmonic oscillator after complexification (Equation (56)).

We can take the inverse of Equations (110) and write the real functions q(t) and p(t) as

(114) $$\begin{array}{ccc} q^{k}\left(t\right) & = & \frac{1}{\sqrt{2}}\left[c_{k}\left(t\right)+c_{k}^{*}\left(t\right)\right]=\frac{1}{\sqrt{2}}\left[Q_{k}\left(t\right)+\frac{1}{𝚤\hbar }P_{k}\left(t\right)\right]\\ p_{k}\left(t\right) & = & \frac{-𝚤}{\sqrt{2}}\left[c_{k}\left(t\right)-c_{k}^{*}\left(t\right)\right]=\frac{-𝚤}{\sqrt{2}}\left[Q_{k}\left(t\right)-\frac{1}{𝚤\hbar }P_{k}\left(t\right)\right]\\ & = & \frac{1}{\sqrt{2}}\left[\frac{1}{\hbar }P_{k}\left(t\right)-𝚤Q_{k}\left(t\right)\right]. \end{array}$$

Adopting the realification of the complex Projective Hilbert space, we may transform Hamilton’s equations to

(115) $$h\left(q,p\right)=\frac{1}{2\hbar }\sum_{k}\sum_{l}\left(q^{k},\;p_{k}\right)h_{kl}\left(\begin{array}{c} q^{l}\\ p_{l} \end{array}\right)$$

(116) $$\begin{array}{ccc} {\dot{q}}^{k} & = & \frac{1}{\hbar }\frac{\partial h(q,p)}{\partial p_{k}}\\ {\dot{p}}_{k} & = & -\frac{1}{\hbar }\frac{\partial h(q,p)}{\partial q^{k}},\;\;k=1,\ldots ,n. \end{array}$$

or

(117) $$\left(\begin{array}{c} \dot{q}\\ \dot{p} \end{array}\right)=\frac{J}{\hbar }\left(\begin{array}{c} \frac{\partial h(q,p)}{\partial q}\\ \frac{\partial h(q,p)}{\partial p} \end{array}\right).$$

$h_{kl}$ is the representation of Hamiltonian operator in the basis set |χ(s)>, and thus, a Hermitian matrix (The Hamiltonian operator is Hermitian, and its representation in a basis results in a Hermitian matrix, $h_{kl}=h_{lk}^{*}$).

The Hamiltonian vector field is extracted from an equation similar to that of classical mechanics (Equation (31)). Hence, in the realified phase space with coordinates (q,p) the ***canonical Poincaré* 1–*form***

(118) $$\theta_{h}=\frac{1}{2\hbar }\sum_{k}\left(p_{k}dq^{k}-q^{k}dp_{k}\right),$$

provides the symplectic 2–form

(119) $$\omega_{h}=-d\theta_{h}=\frac{1}{\hbar }\sum_{k}\left(dq^{k}\wedge dp_{k}\right).$$

Then, the interior product (contraction) of $\omega_{h}$ gives

(120) $$i_{X_{h}}\omega_{h}=\omega_{h}\left(X_{h},•\right)=dh\left(•\right)$$

and the Hamiltonian vector field is extracted as

(121) $$X_{h}=\frac{1}{\hbar }\sum_{k}\left[\left(\frac{\partial h}{\partial p_{k}}\right)\frac{\partial }{\partial q^{k}}-\left(\frac{\partial h}{\partial q^{k}}\right)\frac{\partial }{\partial p_{k}}\right].$$

We define the length of a curve C in the Projective Hilbert space by introducing the Riemannian metric $g(|\dot{\psi }>,|\dot{\psi }>)$ as

(122) $$\begin{array}{ccc} L(C) & = & \int_{0}^{t_{max}}\sqrt{g(|\dot{\psi }>,|\dot{\psi }>)}\;dt=\int_{0}^{t_{max}}\sqrt{<\dot{\psi }|\dot{\psi }>}\;dt\\ & = & \int_{0}^{t_{max}}\sqrt{\sum_{k}{\dot{c}}_{k}^{*}{\dot{c}}_{k}}\;dt=\int_{0}^{t_{max}}\sqrt{\frac{1}{𝚤\hbar }\left(\sum_{k}{\dot{P}}_{k}{\dot{Q}}_{k}\right)}\;dt\\ & = & \frac{1}{2}\int_{0}^{t_{max}}\sqrt{\sum_{k}[{\left({\dot{q}}^{k}\right)}^{2}+{\left({\dot{p}}_{k}\right)}^{2}}]\;dt\\ & = & \frac{1}{2}\int_{0}^{t_{max}}\sqrt{\sum_{k}\left(dq^{k}⊗dq^{k}+dp_{k}⊗dp_{k}\right)\left(\right|\dot{\psi }>,\left|\dot{\psi }>\right)}\;dt. \end{array}$$

For real wavefunction, the length is the Euclidean distance

(123) $$L\left(C\right)=\frac{1}{2}\int_{0}^{t_{max}}\sqrt{\sum_{k}{\left({\dot{q}}^{k}\right)}^{2}}\;dt.$$

#### 2.2.5. Quantum Systems as Totally Integrable Hamiltonian Systems

It has been shown that geometrical quantum mechanics naturally describes a completely integrable system, as follows. Let $H^{n+1}$ be a complex separable Hilbert space of dimension n+1, and view the triple $\left(P^{n}\left(H^{n+1}\right),\omega_{h},X_{h}\right)$ as a Hamiltonian dynamical system on the phase space $P^{n}\left(H^{n+1}\right)$, equipped with the symplectic 2–form $\omega_{h}$. Let $\hat{H}$ be the self-adjoint Hamiltonian operator for the system, and assume that each eigenspace of $\hat{H}$ is one-dimensional (non-degenerate). Choose an orthonormal basis $|\chi_{0}>,\cdots ,|\chi_{n}>$ for $H^{n+1}$ consisting of eigenvectors of $\hat{H}$. Define the projection operators ${\hat{P}}_{0},{\hat{P}}_{1},\cdots ,{\hat{P}}_{n}$ by

(124) $${\hat{P}}_{k}=|\chi_{k}><\chi_{k}|,\;\;\;\;{\hat{P}}_{k}\left(|\psi >\right)=<\chi_{k}\left|\psi >\right|\chi_{k}>=\psi_{k}|\chi_{k}>,$$

where we have used Equation (104).

Without loss of generality, we set the lowest eigenvalue ($w_{0}$) to be 0 so that we can expand the Hamiltonian operator as

(125) $$\hat{H}=\sum_{k=1}^{n}w_{k}{\hat{P}}_{k}.$$

Observe that the projectors $\left\{{\hat{P}}_{k}\right\}$ form a mutually commuting set of *n* operators on $H^{n+1}$, and we can define the corresponding expectation value functions $\left\{P_{k}\right\}$ in the Projective Hilbert space $P^{n}\left(H^{n+1}\right)$ as

(126) $$P_{k}:P^{n}\left(H\right)\to R,\;\;P_{k}\left(\left\{|\psi >\right\}\right)=\frac{<\psi |{\hat{P}}_{k}|\psi >}{<\psi |\psi >}=<\psi |{\hat{P}}_{k}\left|\psi >=\right|\psi_{k}{|}^{2}.$$

Hence,

(127) $$<\psi |{\hat{H}}^{n+1}|\psi >=\sum_{k=1}^{n}w_{k}{\left|\psi_{k}\right|}^{2}=\sum_{k=1}^{n}w_{k}\left(q^{k2}+p_{k}^{2}\right).$$

$\left(q^{k},p_{k}\right)$ are the real and imaginary parts of the wavefunction. Thus, the Hamiltonian in the projective Hilbert space resembles that of a sum of *n* harmonic oscillators in scaled canonical coordinates (Equation (55)).

Therefore, we may conclude that there are *n* constants of motion, $P_{k}$, and the trajectories lie on n–dimensional tori (Lagrangian tori).

## 3. Hamiltonian Chemical Thermodynamics

### 3.1. Manifolds and Maps

**Figure 4:**
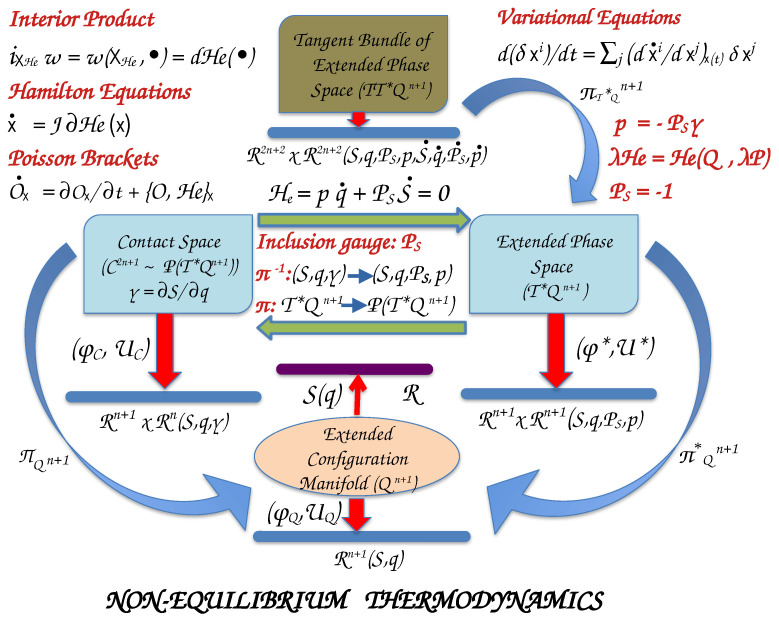
Manifolds and functions which determine the geometrical structures of a thermodynamical system with n+1 coordinates. *S* denotes the entropy and $q={\left(q^{1},\ldots ,q^{n}\right)}^{T}$ are the coordinates of n–extensive properties. γ are the partial derivatives of entropy that correspond to the intensive properties of the system. With the inclusion of gauge $P_{S}$, the conjugate momenta *p* are defined with respect to which homogeneous Hamiltonians, $H_{e}$, of first-degree are determined. Details are given in the text.

To establish an analogous theoretical framework for classical thermodynamics to that of Hamiltonian classical mechanics, we consider the macroscopic properties of the system to consist of the configuration manifold (Figure 4). For a typical chemical system we usually take the ***extensive*** physical properties of entropy (*S*), internal energy (*U*), volume (*V*), and the number of molecules (or moles) ($N^{1},\ldots ,N^{M}$) of *M* chemical species to consist of the coordinates of the extended configuration manifold $Q^{n+1}$, where n=2+M. If we attribute entropy to be a homogeneous first-degree function of $q={\left(q^{1},q^{2},\ldots ,q^{n}\right)}^{T}\equiv {\left(U,V,N^{1},\ldots ,N^{M}\right)}^{T}\in Q^{n+1}$, then according to Euler’s theorem for homogeneous functions we can write

(128) $$\begin{array}{ccc} S(U,V,N) & = & {\left(\frac{\partial S}{\partial U}\right)}_{V,N}U+{\left(\frac{\partial S}{\partial V}\right)}_{U,N}V+\sum_{i=1}^{M}{\left(\frac{\partial S}{\partial N^{i}}\right)}_{U,V,N^{j\neq i}}N^{i},\;\;j=1,\ldots ,M\\ & = & \frac{1}{T}U+\frac{P}{T}V-\sum_{i=1}^{M}\frac{\mu_{i}}{T}N^{i}=\gamma_{1}U+\gamma_{2}V+\sum_{k=3}^{n}\gamma_{k}N^{k-2}, \end{array}$$

where we have introduced the ***conjugate intensive*** variables $\left\{\gamma_{i}\right\}$ as the partial derivatives of entropy

$$\gamma_{1}=\frac{\partial S}{\partial U}=\frac{1}{T},\;\;\gamma_{2}=\frac{\partial S}{\partial V}=\frac{P}{T},\;\;\gamma_{k}=\frac{\partial S}{\partial N^{k-2}}=-\frac{\mu_{k-2}}{T},\;\;k=3,\ldots ,n.$$

*T* is the absolute temperature, *P* the pressure, and $\mu_{1},\ldots ,\mu_{M}$ the chemical potentials of *M* compounds.

(q,γ) and entropy comprise of a (2n+1)D–coordinate system for the ***contact manifold*** $\left(C^{2n+1}\right)$, where the physical states of the system live. The extended configuration manifold and the contact manifold are generally nonlinear, and the maps $\left(\phi_{Q},U_{Q}\right)$ and $\left(\phi_{C},U_{C}\right)$ determine local coordinate systems in Euclidean space.

The total differential of *S* is Gibbs’s fundamental equation that describes the ***physical thermodynamic submanifold*** (PTS) of a thermodynamic system

(129) $$dS=\frac{1}{T}dU+\frac{P}{T}dV-\sum_{i=1}^{M}\frac{\mu_{i}}{T}dN^{i}.$$

If we assign the entropy *S* to coordinate $q^{0}$, we can also write

(130) $$dq^{0}=\sum_{k=1}^{n}\gamma_{k}\left(q\right)dq^{k}.$$

From Equations (129) and (130) we deduce that the 1–form $\theta_{c}$ satisfies

(131) $$\theta_{c}=dS-\frac{1}{T}dU-\frac{P}{T}dV+\sum_{i=1}^{M}\frac{\mu_{i}}{T}dN^{i}=dq^{0}-\sum_{k=1}^{n}\gamma_{k}\left(q\right)dq^{k}=0.$$

This equation expresses the physical state submanifold of a thermodynamic system, named ***Legendrian submanifold***, and it is what we represent with the equation of states [32].

In a more formal wording, in the contact space $C^{2n+1}$ the locally defined 1–form $\theta_{c}$ that satisfies the condition, i.e., there is the volume form

$$\theta_{c}\wedge {\left(d\theta_{c}\right)}^{n}\neq 0,$$

ascertains the nD–Legendrian submanifold $L_{c}^{n}$ of $C^{2n+1}$ by requiring $\theta_{c}=0$. In other words, the ***kernel*** of $\theta_{c}$ provides the maximal dimension hyperplanes tangent to $L_{c}^{n}$.

To construct a Hamiltonian system, we need to define the canonical conjugate momenta. Following Balian and Valentine [18], we denote the conjugate momentum of entropy with $P_{S}$, and we take it as a non-vanishing free parameter or a gauge variable. Then, the canonical conjugate momenta to the other coordinates of $Q^{n+1}$ are defined as

(132) $$p_{1}=-P_{S}\gamma_{1},\;\;p_{2}=-P_{S}\gamma_{2},\;\;p_{3}=-P_{S}\gamma_{3},\;\;\cdots ,p_{n}=-P_{S}\gamma_{n}.$$

Hence, $\left\{p_{i}\right\}$ act as new variables, which replace the physical intensive variables $\left\{\gamma_{i}\right\}$. We write $P_{S}=p_{0}$ and Equation (131) determines the Poincaré 1–form

(133) $$\theta_{e}=p_{0}dq^{0}+\sum_{k=1}^{n}p_{k}dq^{k}=\sum_{k=0}^{n}p_{k}dq^{k}=0,$$

which produces the symplectic 2–form

(134) $$\omega_{e}=-d\theta_{e}=\sum_{k=0}^{n}dq^{k}\wedge dp_{k}=0.$$

This equation sets the ***Thermodynamic Extended Physical State Submanifold (TEPSS)*** in the ***extended phase space***, $T^{*}Q^{n+1}\equiv P^{2n+2}$, named ***Lagrangian submanifold***.

In a more formal wording, for an even-dimensional phase space, $P^{2n+2}$, the symplectic 2–form $\omega_{e}$ (see also Equation (29)), which satisfies the condition (volume form)

$${\left(d\theta_{e}\right)}^{n+1}\neq 0,$$

determines the (n+1)D–Lagrangian submanifold $L_{p}^{n+1}$ in $P^{2n+2}$ by requiring $\omega_{e}=0$.

The thermodynamic contact space $C^{2n+1}$ is the ***projective space*** of thermodynamic extended phase space $P^{2n+2}$ (see Appendix A.4.1)

(135) $$\pi :T^{*}Q^{n+1}\to P^{2n+1}\left(T^{*}Q^{n+1}\right),$$

where π is the projection map.

It is proved that the submanifold $L_{c}^{n}\subset P^{2n+1}\left(T^{*}Q^{n+1}\right)$ is a Legendrian submanifold, if and only if

$$L_{p}^{n+1}:=\pi^{-1}\left(L_{c}^{n}\right)\subset T^{*}Q^{n+1}$$

is a ***homogeneous in momenta Lagrangian submanifold*** (see Table A1). This means that $\left(d\theta_{e}=0\right)$ as well as

(136) $$\left(Q,P\right)\in L_{p}^{n+1}\Rightarrow \left(Q,\lambda P\right)\in L_{p}^{n+1},\;\;\mathrm{for}\;\;\mathrm{every}\;\;\lambda \neq 0.$$

We say that all $Q=\left(q_{0},q\right),P=\left(p_{0},p\right)$, which satisfy Equation (136), belong to the ***ray*** {Q,P}. Then, it is valid $\theta_{e}=0$ for every vector field tangent to $L_{p}^{n+1}$. Also, every ***homogeneous Lagrangian submanifold*** originates from a Legendrian submanifold of the form $\pi^{-1}\left(L_{c}^{n}\right)$. We can also state that a submanifold is homogeneous if its generating function is homogeneous.

In the entropy representation, the *n* extensive independent variables $\left(q^{1},\ldots ,q^{n}\right)$ together with the $p_{0}$ parameterize the Lagrangian submanifold. If the entropy $S(q^{1},\ldots ,q^{n})$ is the generating function of the Legendrian submanifold, then for the Lagrangian submanifold the generating function is $-p_{0}S\left(q^{1},\ldots ,q^{n}\right)$. This is a property of homogeneous functions of first-degree. Similarly, in energy representation we take $-p_{1}U\left(q^{0},q^{2},\ldots ,q^{n}\right)$ as generating function for the Lagrangian submanifold [29]. With the generating function in the entropy representation, we extract the remaining n+1 variables as

(137) $$q^{0}=S\left(q^{1},\ldots ,q^{n}\right),\;\;\;\;p_{i}=-p_{0}\frac{\partial S}{\partial q^{i}}=-p_{0}\gamma_{i},\;\;i=1,\ldots ,n.$$

The extended Hamiltonian $H_{e}$ is a function on phase space to real numbers, whereas the tangent bundle of phase space, $TT^{*}Q^{n+1}$, is described by coordinates and momenta, $x={\left(Q,P^{T}\right)}^{T}$, as well as their time derivatives. The extended Hamiltonian is a homogeneous function of first-degree in momenta, i.e.,

(138) $$\lambda H_{e}=H_{e}\left(Q,\lambda P\right).$$

$\pi_{T^{*}Q^{n+1}}$ is the projection map of the tangent bundle of phase space to phase space.

In Appendix A.4, we summarize in Table A2 and Table A3 several formulae of Hamiltonian thermodynamics in entropy- and energy-representations, respectively.

### 3.2. Equations of Motion

The Hamiltonian function $H_{e}\left(Q,P\right)$ acting in the extended phase space is a homogeneous function of first-degree in momenta [18] and according to Euler’s theorem for homogeneous functions of first-degree, we can write

(139) $$H_{e}=\sum_{i=0}^{n}p_{i}\frac{\partial H_{e}}{\partial p_{i}}.$$

For a simple thermodynamic system, the extended Hamiltonian is $H_{e}=p\dot{q}+P_{S}\dot{S}$. To extract the physical states, we impose the constrain $P_{S}=-1$, which implies $H_{e}=0$ on thermodynamic extended physical state submanifold, also written as [29]

(140) $$H_{e}=\sum_{i=1}^{n}\left(p_{i}+p_{0}\frac{\partial S}{\partial q^{i}}\right){\dot{q}}^{i}=p\dot{q}+P_{S}\dot{S}=0.$$

We point out that a Hamiltonian system is the triple $\left(P^{2n+2},\omega_{e},X_{H_{e}}\right)$, where $X_{H_{e}}$ is the Hamiltonian vector field defined on the tangent bundle of phase space, and $\omega_{e}$ a skew-symmetric, non-degenerate, closed differential 2–form. Then, we can use the machinery of geometrical classical mechanics as was developed in Section 2.1 to write down equations for equilibrium and non-equilibrium processes. Thus, the Hamiltonian vector field can be extracted from the equation

(141) $$i_{X_{H_{e}}}\omega_{e}\left(X_{H_{e}},•\right)=dH_{e}\left(•\right),$$

given the Hamiltonian function $H_{e}$ on the phase space $P^{2n+2}$. This equation provides the Hamiltonian vector field $X_{H_{e}}$

(142) $$X_{H_{e}}=\sum_{k=0}^{n}{\left(\frac{\partial H_{e}}{\partial p_{k}}\frac{\partial }{\partial q^{k}}-\frac{\partial H_{e}}{\partial q^{k}}\frac{\partial }{\partial p_{k}}\right)}^{T}.$$

From the definition of the extended Poincaré 1–form (Equation (133)) we prove that

(143) $$\theta_{e}\left(X_{H_{e}}\right)=H_{e},$$

for homogeneous in momenta of first-degree Hamiltonian functions.

Hence, Hamilton’s equations take their usual form (Equation (33))

(144) $${\dot{q}}^{i}=\frac{\partial H_{e}}{\partial p_{i}},\;\;\;\;{\dot{p}}_{i}=-\frac{\partial H_{e}}{\partial q^{i}},\;\;i=0,\ldots ,n.$$

Moreover, (Q,P) is a canonical coordinate system in the corresponding phase space, and thus, satisfy the ***Poisson brackets Equation (34)***

(145) $$\begin{array}{ccc} \left\{q^{i},q^{j}\right\}=\left\{p_{i},p_{j}\right\} & = & 0, \end{array}$$

(146) $$\begin{array}{ccc} \left\{q^{i},p_{j}\right\} & = & \delta_{j}^{i}, \end{array}$$

(147) $$\begin{array}{ccc} {\dot{q}}^{i}=\left\{q^{i},H_{e}\right\},\;\;\;{\dot{p}}_{j} & = & \{p_{j},H_{e}\}. \end{array}$$

#### 3.2.1. Contact Equations of Motion

Since the Poisson bracket (Equation (34)) is invariant under gauge (canonical) transformations, we obtain

(148) $$H_{e}\left(q^{0},q^{1},\ldots ,q^{n},p_{0},p_{1},\ldots ,p_{n}\right)=-p_{0}F\left(q^{0},q^{1},\ldots ,q^{n},\gamma_{1},\ldots ,\gamma_{n}\right),$$

where *F* is a generating function and

(149) $$\gamma_{i}=-\frac{p_{i}}{p_{0}},\;\;\;\;i=1,\ldots ,n,$$

we extract the contact vector field (see Appendix A.3)

(150) $$\begin{array}{ccc} \dot{q^{0}} & = & X_{c}^{0}=-F+\sum_{i=1}^{n}\gamma_{i}\frac{\partial F}{\partial \gamma_{i}} \end{array}$$

(151) $$\begin{array}{ccc} \dot{q^{i}} & = & X_{c}^{i}=\frac{\partial F}{\partial \gamma_{i}},\;\;\;\;i=1,\ldots ,n \end{array}$$

(152) $$\begin{array}{ccc} {\dot{\gamma }}_{i} & = & {\bar{X}}_{c}^{i}=-\left(\frac{\partial F}{\partial q^{i}}+\gamma_{i}\frac{\partial F}{\partial q^{0}}\right),\;\;\;\;i=1,\ldots ,n. \end{array}$$

For a generating function *F*, which is independent of $q_{0}$ variable, as well as a homogeneous function of first-degree in momenta, the contact equations of motion are reduced to those of Hamilton’s equations in $P^{2n}$ phase space, i.e.,

(153) $$\dot{q^{0}}=0,\;\;\;\;\dot{q^{i}}=\frac{\partial F}{\partial \gamma_{i}},\;\;\;\;{\dot{\gamma }}_{i}=-\frac{\partial F}{\partial q^{i}},\;\;\;\;i=1,\ldots ,n.$$

#### 3.2.2. Riemannian Metric on Lagrangian Submanifold

We can define a Riemannian metric [28] on a Lagrangian (n+1)D–submanifold, $L_{p}^{n+1}\subset P^{2n+2}$, with generating function $-p_{0}S\left(q^{1},\ldots ,q^{n}\right)$. In canonical coordinates (q,p) it takes the form (see Table A2)

(154) $$R_{S}\left(q,p\right)=\frac{1}{p_{0}}\sum_{i=0}^{n}dq^{i}dp_{i}=-\sum_{i=1}^{n}dq^{i}d\gamma_{i}=-\sum_{i=1}^{n}\sum_{j=1}^{n}\frac{\partial^{2}S}{\partial q^{i}\partial q^{j}}dq^{i}dq^{j}.$$

$R_{S}$ is the metric introduced by Ruppeiner [33,34] and it is usually called Ruppeiner metric.

Weinhold [35,36] has extracted a metric, $R_{U}$, in the energy representation of thermodynamics with generating function $-p_{1}U\left(q^{0},q^{2},\ldots ,q^{n}\right)$ [29] (see Table A3).

### 3.3. Embedding Systems in Homogeneous Media

We examine the case of a simple system with internal energy *U* and constant volume and number of particles. The embedded mechanical system is described by the Hamiltonian $H_{d}\left(\sigma ,\pi \right)$. Here, we consider the augmented system, system plus environment, isolated with total energy *E* constant, $\dot{E}=0$

(155) $$E=U+H_{d}\left(\sigma ,\pi \right).$$

The entropy of the thermodynamic system is given by the equation

(156) $$S_{U}=S_{U}\left(U\right)=S_{U}\left(E-H_{d}\left(\sigma ,\pi \right)\right)=S\left(E,\sigma ,\pi \right),$$

i.e., a function of n=2w+1 independent variables. In the extended thermodynamic phase space, we include entropy as an independent variable as well

$$q^{0}=S,\;\;\;\;q^{1}=E,\;\;\;\;q^{2}=\sigma^{1},\ldots ,q^{w+1}=\sigma^{w},\;\;\;\;q^{w+2}=\pi_{1},\ldots ,q^{2w+1}=\pi_{w}.$$

The canonical conjugate momenta are $p_{0}=p_{S},p_{1}=p_{E}$ and $p_{i}=p_{\sigma^{i-1}},i=2,\ldots ,w+1,p_{j}=p_{\pi_{j-(w+1)}},\;\;j=w+2,\ldots ,2w+1$. Hence, the dimension of thermodynamic extended phase space is (2n+2=4w+4)D.

In the entropy representation, we take as the generating function of the Legendrian submanifold in the thermodynamic contact space the entropy $S(q^{1},\ldots ,q^{n})$. Hence, the generating function of the corresponding Lagrangian submanifold in the extended phase space is $-p_{0}S\left(q^{1},\ldots ,q^{n}\right)$. For $p_{0}=-1$ the canonical momenta become

(157) $$p_{i}=\frac{\partial S}{\partial q^{i}}=\gamma_{i},\;\;i=1,\ldots ,n.$$

Therefore, we have attained the important conclusion that the thermodynamic extended state manifold in thermodynamic extended phase space is the Lagrangian submanifold, $L_{p}^{n+1}$,

$$\begin{array}{ccc} L_{p}^{n+1} & = & \left\{x={\left(q,p^{T}\right)}^{T}\in P^{2n+2}\right\} \end{array}$$

(158) $$\begin{array}{ccc} q^{0} & = & S\left(q^{1},q^{2},\ldots ,q^{n}\right)=S\left(E,\sigma^{1},\ldots ,\sigma^{w},\pi_{1},\ldots ,\pi_{w}\right) \end{array}$$

(159) $$\begin{array}{ccc} p_{1} & = & -p_{0}\gamma_{1}=-p_{0}\frac{\partial S}{\partial E} \end{array}$$

(160) $$\begin{array}{ccc} p_{k} & = & -p_{0}\gamma_{k}=-p_{0}\frac{\partial S}{\partial \sigma^{k-1}},\;\;k=2,\ldots ,w+1 \end{array}$$

(161) $$\begin{array}{ccc} p_{l} & = & -p_{0}\gamma_{l}=-p_{0}\frac{\partial S}{\partial \pi_{l-(w+1)}},\;\;l=w+2,\ldots ,2w+1. \end{array}$$

From Equation (140) the Hamiltonian function in the extended phase space is written as

(162) $$\begin{array}{ccc} H_{e} & = & \sum_{i=1}^{n}\left(p_{i}+p_{0}\frac{\partial S}{\partial q^{i}}\right){\dot{q}}^{i}=\left(p_{1}+p_{0}\frac{\partial S}{\partial E}\right)\dot{E}\\ & + & \left(p_{2}+p_{0}\frac{\partial S}{\partial \sigma^{1}}\right){\dot{\sigma }}^{1}+\cdots +\left(p_{w+1}+p_{0}\frac{\partial S}{\partial \sigma^{w}}\right){\dot{\sigma }}^{w}\\ & + & \left(p_{w+2}+p_{0}\frac{\partial S}{\partial \pi_{1}}\right){\dot{\pi }}_{1}+\cdots +\left(p_{2w+1}+p_{0}\frac{\partial S}{\partial \pi_{w}}\right){\dot{\pi }}_{w} \end{array}$$

Hamilton’s equations are then determined

(163) $$\begin{array}{ccc} {\dot{q}}^{0} & = & \frac{\partial H_{e}}{\partial p_{0}}=\sum_{i=1}^{n}\left(\frac{\partial S}{\partial q^{i}}\right){\dot{q}}^{i} \end{array}$$

(164) $$\begin{array}{ccc} {\dot{q}}^{1} & = & \frac{\partial H_{e}}{\partial p_{1}}=\dot{E}=0 \end{array}$$

(165) $$\begin{array}{ccc} {\dot{q}}^{j} & = & \frac{\partial H_{e}}{\partial p_{j}}={\dot{x}}_{d}^{j-1},\;\;j=2,\ldots ,n, \end{array}$$

(166) $$\begin{array}{ccc} {\dot{p}}_{0} & = & -\frac{\partial H_{e}}{\partial q^{0}}=0 \end{array}$$

(167) $$\begin{array}{ccc} {\dot{p}}_{1} & = & -\frac{\partial H_{e}}{\partial q^{1}}=-\sum_{i=1}^{n}p_{0}\left(\frac{\partial^{2}S}{\partial q^{1}\partial q^{i}}\right){\dot{q}}^{i}-\sum_{i=1}^{n}\left(p_{i}+p_{0}\frac{\partial S}{\partial q^{i}}\right)\frac{\partial {\dot{q}}^{i}}{\partial q^{1}} \end{array}$$

(168) $$\begin{array}{ccc} {\dot{p}}_{j} & = & -\frac{\partial H_{e}}{\partial q^{j}}=-\sum_{i=1}^{n}p_{0}\left(\frac{\partial^{2}S}{\partial q^{j}\partial q^{i}}\right){\dot{q}}^{i}-\sum_{i=1}^{n}\left(p_{i}+p_{0}\frac{\partial S}{\partial q^{i}}\right)\frac{\partial {\dot{q}}^{i}}{\partial q^{j}},\;\;j=2,\ldots ,n, \end{array}$$

where the velocities ${\dot{x}}_{d}^{j-1}$ are determined from the definition of the embedded system.

### 3.4. Chemical Kinetics

The kinetics of chemical reaction networks can also be formulated using a geometric Hamiltonian theory as an embedded system. However, classical chemical kinetics is better studied by introducing temperature, pressure, and the elementary chemical reaction coordinates (ξ) as the independent variables.

#### 3.4.1. Thermodynamics of Chemical Reactions

A general chemical reaction with r–reactant compounds and p–product compounds can be expressed by the formula

(169) $$0=\sum_{j=1}^{M}\nu_{j}J_{j},$$

where M=r+p. The stoichiometric integers $\nu_{j},j=1,\ldots ,r$ are negative for the reactant molecules $\left\{J_{j}\right\}=\left(A_{1},\ldots ,A_{r}\right)$ and positive $\nu_{j},j=r+1,\ldots ,M$, for the product molecules, $\left\{J_{j}\right\}=\left(B_{1},\ldots ,B_{p}\right)$.

For *K* elementary chemical reactions, we must employ *K* independent reaction coordinates $\xi^{1},\ldots ,\xi^{K}$. Then, the time variation of the number of molecules is related to the velocities of reactions by the equation

(170) $$\left(\begin{array}{c} {\dot{N}}^{1}\\ {\dot{N}}^{2}\\ \ldots \\ {\dot{N}}^{M} \end{array}\right)=\left(\begin{array}{cccc} \nu_{11} & \nu_{12} & \ldots & \nu_{1K}\\ \nu_{21} & \nu_{22} & \ldots & \nu_{2K}\\ \ldots & \ldots & \ldots & \ldots \\ \nu_{M1} & \nu_{M2} & \ldots & \nu_{MK} \end{array}\right)\left(\begin{array}{c} {\dot{\xi }}^{1}\\ {\dot{\xi }}^{2}\\ \ldots \\ {\dot{\xi }}^{K} \end{array}\right),$$

or simply as

(171) $$\dot{N}=C\dot{\xi },$$

where $N={\left(N^{1},\ldots ,N^{M}\right)}^{T}\equiv {\left(N_{A_{1}},\ldots ,N_{A_{r}},N_{B_{1}},\ldots ,N_{B_{p}}\right)}^{T}$ and $C=[\nu_{jk}],j=1,\ldots ,M,$k=1,…,K, being the ***stoichiometric matrix*** for the *K* elementary reactions.

The reaction Gibbs free energy $\Delta_{r}G$ is defined by introducing the chemical potentials of the $\left\{J_{j}\right\}$ compounds

(172) $$\mu_{j}={\left(\frac{\partial G}{\partial N^{j}}\right)}_{T,P,N^{i\neq j}},\;\;\;\;i,j=1,\ldots ,M,$$

where $G(T,P,N^{1},\ldots ,N^{M})$ is Gibbs free energy, *T* the temperature and *P* the pressure. From Gibbs’s fundamental equation

(173) $$dG\left(T,P,N\right)=-SdT+VdP+\sum_{j=1}^{M}\mu_{j}dN^{j},$$

and for a general chemical reaction at constant temperature and pressure, Equation (169), we have

(174) $$dG=\sum_{j=1}^{M}\mu_{j}\left(T,P\right)dN^{j}=\sum_{j=1}^{M}\mu_{j}\left(T,P\right)\left(\nu_{j}d\xi \right).$$

Therefore, the above equation becomes

(175) $$\left(\frac{dG}{d\xi }\right)=\sum_{j=1}^{M}\nu_{j}\mu_{j}\left(T,P\right).$$

Usually, the derivative of Gibbs free energy with the reaction coordinate is denoted as $\Delta_{r}G_{m}=\sum_{j=1}^{M}\nu_{j}\mu_{j}\left(T,P\right)$ and is called ***molar reaction Gibbs free energy***.

For *K* elementary reactions, we write

(176) $${\dot{\xi }}^{k}=R_{fk}-R_{bk},\;\;\;\;k=1,\ldots ,K,$$

employing the rates of forward ($R_{fk}$) and backward ($R_{bk}$) reactions, as expressed by the law of Mass Action [37,38,39,40].

Also, for an elementary chemical reaction De Donder [41,42] introduced the quantity of ***Affinity***

(177) $$A=-\Delta_{r}G_{m}=-\sum_{j=1}^{M}\nu_{j}\mu_{j},$$

which can be written as [30]

(178) $$A_{\xi^{k}}=RT\ln\left(\frac{R_{fk}\left(\xi^{k}\right)}{R_{bk}\left(\xi^{k}\right)}\right).$$

#### 3.4.2. Thermodynamic Hamiltonian in Massieu-Gibbs Representation

Chemical reactions are better studied at constant temperature and pressure. Hence, we first transform the entropy from a function of the internal energy and volume to a function in temperature and pressure by a Legendre transformation

(179) $$L:{\left(S,U,V,N\right)}^{T}\to {\left(S,T,P,N\right)}^{T},$$

(180) $$LS\left[\frac{1}{T},\frac{P}{T}\right]\equiv S=S-\frac{1}{T}U-\frac{P}{T}V=-\frac{G(T,P,N)}{T}.$$

S is Massieu function related to Gibbs free energy G(T,P,N)=U−TS+PV [19].

The above Legendre transformation is a diffeomorphism on the Legendrian and Lagrangian submanifolds. This means that we can define Affinity with the chemical potentials $\mu_{j}$ of *M* chemical compounds (reactants and products) given by Gibbs free energy

(181) $$\mu_{j}={\left(\frac{\partial G}{\partial N^{j}}\right)}_{T,P,N^{i\neq j}},\;\;i,j=1,\ldots ,M.$$

The rate of entropy production for the *k*th-irreversible reaction is written with the molar reaction Gibbs free energy $\left(\Delta_{r}G_{m,k}\right)$

(182) $$\frac{dS_{k}}{dt}=-\frac{\Delta_{r}G_{m,k}}{T}\frac{d\xi_{k}}{dt}=\frac{A_{\xi^{k}}}{T}{\dot{\xi }}^{k},$$

where $A_{\xi^{k}}$ is the Affinity of *k*th-reaction and $\xi^{k}$ is the reaction coordinate that describes the progress (extent) of the reaction. The total entropy rate for *K* reactions becomes

(183) $$\frac{dS}{dt}=\sum_{k=1}^{K}\frac{A_{\xi^{k}}}{T}\frac{d\xi^{k}}{dt}\geq 0,$$

or employing Equations (176) and (178), as

(184) $$\frac{dS}{dt}=R\sum_{k=1}^{K}\left[R_{fk}\left(t\right)-R_{bk}\left(t\right)\right]\ln\left(\frac{R_{fk}\left(t\right)}{R_{bk}\left(t\right)}\right),$$

where *R* is the ideal gas constant. Obviously, for reactions at equilibrium in a closed thermodynamic system, we have $R_{fk}=R_{bk}$, which means that $\frac{dS}{dt}=0$, and thus, the total entropy is conserved. The inequality in Equation (183), which is consistent with the second thermodynamic law, holds because ln is a monotonous function in the domain of its definition.

The thermodynamic extended state manifold in thermodynamic extended phase space is a Lagrangian submanifold, $L_{p}^{n+1}$ embedding in $P^{2n+2}$ phase space with n=2+M. The generating function is $-p_{0}S=p_{0}G\left(T,P,N\right)/T$

(185) $$\begin{array}{ccc} L_{p}^{n+1} & = & \left\{{\left(q,p\right)}^{T}\in P^{2n+2}\right\} \end{array}$$

(186) $$\begin{array}{ccc} q^{0} & = & S(T,P,N^{1},\ldots ,N^{M}) \end{array}$$

(187) $$\begin{array}{ccc} p_{1} & = & -p_{0}\frac{\partial S}{\partial T}\\ p_{2} & = & -p_{0}\frac{\partial S}{\partial P}\\ p_{k} & = & -p_{0}\frac{\partial S}{\partial N^{k-2}},\;\;k=3,\ldots ,n. \end{array}$$

$p_{0}$ is the conjugate momentum of S.

The Hamiltonian function in the extended cotangent bundle (phase space) is written as

(188) $$\begin{array}{ccc} H_{e}^{S} & = & {\langle \left(p+p_{0}\frac{\partial S}{\partial q}\right)|\dot{q}\rangle }_{n}=\sum_{i=1}^{n}\left(p_{i}+p_{0}\frac{\partial S}{\partial q^{i}}\right){\dot{q}}^{i}\\ & = & \left(p_{1}+p_{0}\frac{\partial S}{\partial T}\right)\dot{T}+\left(p_{2}+p_{0}\frac{\partial S}{\partial P}\right)\dot{P}+\\ & & \left(p_{3}-p_{0}\frac{\mu_{1}}{T}\right){\dot{N}}^{1}+\ldots +\left(p_{n}-p_{0}\frac{\mu_{M}}{T}\right){\dot{N}}^{M}. \end{array}$$

At constant temperature and pressure and using concentrations instead of the number of molecules for reactants and products, ${\left(x_{1},\ldots ,x_{M}\right)}^{T}$ with conjugate momenta also denoted by $\left(p_{1},\ldots ,p_{M}\right)$, we take

(189) $$H_{e}^{S}=\left(p_{1}-p_{0}\frac{\mu_{1}}{T}\right){\dot{x}}_{1}+\cdots +\left(p_{M}-p_{0}\frac{\mu_{M}}{T}\right){\dot{x}}_{M}.$$

For *K* elementary reactions in the reaction coordinate space, $\xi ={\left(\xi^{1},\ldots ,\xi^{K}\right)}^{T}$, and under the transformation of stoichiometric matrix C:ξ→x we have

(190) $$\dot{x}=C\dot{\xi },\;\;\;\;\;p_{\xi }=pC,\;\;\;\;\;A_{\xi }=\mu C,$$

and the Hamiltonian (Equation (189)) becomes

(191) $$H_{\xi }^{S}=\sum_{k=1}^{K}\left(p_{\xi^{k}}+\frac{p_{0}}{T}A_{\xi^{k}}\right){\dot{\xi }}^{k}.$$

Then, Hamilton’s equations are written

(192) $$\begin{array}{ccc} \dot{S} & = & \frac{\partial H_{\xi }^{S}}{\partial p_{0}}=\sum_{k=1}^{K}\frac{A_{\xi^{k}}}{T}{\dot{\xi }}^{k}=R\sum_{k=1}^{K}\left[R_{fk}\left(\xi \right)-R_{bk}\left(\xi \right)\right]\ln\left(\frac{R_{fk}\left(\xi \right)}{R_{bk}\left(\xi \right)}\right)\\ {\dot{\xi }}^{k} & = & \frac{\partial H_{\xi }^{S}}{\partial p_{\xi^{k}}}=R_{fk}\left(\xi \right)-R_{bk}\left(\xi \right),\;\;\;\;k=1,\ldots ,K\\ {\dot{p}}_{0} & = & -\frac{\partial H_{\xi }^{S}}{\partial S}=0\\ {\dot{p}}_{\xi^{i}} & = & -\frac{\partial H_{\xi }^{S}}{\partial \xi^{i}}=-\frac{p_{0}}{T}\sum_{k=1}^{K}\left(\frac{\partial A_{\xi^{k}}}{\partial \xi^{i}}\right){\dot{\xi }}^{k}-\sum_{k=1}^{K}\left(p_{\xi^{k}}+\frac{p_{0}}{T}A_{\xi^{k}}\right)\frac{\partial {\dot{\xi }}^{k}}{\partial \xi^{i}},\;\;i=1,\ldots ,K. \end{array}$$

We can also define a metric on the Lagrangian submanifold in Massieu-Gibbs representation, and this is given as

(193) $$dl^{2}=\frac{1}{p_{0}}\sum_{i=1}^{K}dp_{\xi^{i}}d\xi^{i}=-\sum_{i=1}^{K}\sum_{k=1}^{K}\frac{\partial^{2}S}{\partial \xi^{i}\partial \xi^{k}}d\xi^{i}d\xi^{k}=\frac{1}{T}\sum_{i=1}^{K}\sum_{k=1}^{K}\frac{\partial^{2}G}{\partial \xi^{i}\partial \xi^{k}}d\xi^{i}d\xi^{k}.$$

Therefore, to measure the distance between initial and equilibrium states, we compute the length of a path on the thermodynamic extended state manifold

(194) $$L\left(t_{max}\right)=\int_{0}^{t_{max}}\sqrt{-\sum_{i=1}^{K}{\dot{p}}_{\xi^{i}}{\dot{\xi }}^{i}}\;\;dt,$$

with $t_{max}$ to be the maximum integration time and substituting $p_{0}=-1$. The distance of two states has been utilized to define a better low bound of the entropy production or dissipated work (availability) than zero for finite time irreversible processes [43,44].

In Appendix A.5, an example of two elementary consecutive chemical reactions is presented and described in detail.

## 4. Numerical Implementations

### 4.1. High-Order Finite-Difference and Pseudospectral Methods

High-order finite-difference methods are widespread for solving the differential equations encountered in classical and quantum mechanics. They are based on polynomial approximations to the solution functions, and thus, finite-difference methods (FD) are suited for problems that can be solved by expanding the unknown functions to Taylor series [25,45]. In the past, we developed and tested high-order multivariable finite-difference methods in configuration space and time (herein they are denoted with *x*), formulated by ***Lagrange interpolating polynomials*** [46,47]. Given that variable order finite-difference algorithms are among the most suitable for modern high-performance computing, the computer technology to which computational chemistry is mainly addressed, as well as their programming simplicity, finite-difference methods emerge as one of the best choices for studying chemical dynamics.

There is a huge literature concerning finite-difference approximations to initial value problems (Notice that Hamilton’s equations, Jacobi fields, and Poisson brackets can be written as initial value problems, and thus, ordinary differential equations can simultaneously be integrated.), such as Hamilton’s and variational equations [45], as well as calculating the action of Hamiltonian operator on wavefunctions in quantum mechanics [25].

For ordinary differential equations (ODEs) and partial differential equations, we approximate the solution functions by expanding them in an appropriate basis set $\left\{\chi_{j}\left(x\right)\right\}$

(195) $$\psi \left(x\right)\approx \psi_{N}\left(x\right)=\sum_{j=1}^{N}a_{j}\chi_{j}\left(x\right).$$

Different global basis functions produce different pseudospectral methods. From such so-called finite basis representation, we can transform to a ***cardinal set of basis functions***, $\left\{u_{j}\left(x\right)\right\}$, also called discrete variable representation, by choosing *N* grid points, $\left\{x_{i}\right\}$, at which the function is calculated. The cardinal functions obey the δ–Kronecker property

(196) $$u_{j}\left(x_{k}\right)=\delta_{kj},$$

so that the wavefunction is expressed by the set of grid points

(197) $$\psi_{N}\left(x\right)=\sum_{j=1}^{N}\psi \left(x_{j}\right)u_{j}\left(x\right).$$

The transformation from $\chi_{j}\left(x\right)$ to the cardinal basis set is unitary, and the new basis is given in terms of the old one by the equation

(198) $$<\chi_{i}|u_{j}>=\sum_{k=1}^{N}w_{k}\chi_{i}^{*}\left(x_{k}\right)u_{j}\left(x_{k}\right)=w_{j}\chi_{i}^{*}\left(x_{j}\right),$$

where the grid points $x_{k}$ and the corresponding weights $w_{k}$ depend on the chosen quadrature rule.

The *m*th-derivative of the approximate solution is written as

(199) $${\left.\frac{d^{m}\psi_{N}\left(x\right)}{dx^{m}}\right|}_{x=x_{k}}=\sum_{j=1}^{N}b_{k,j}^{\left(m\right)}\psi \left(x_{j}\right)=D^{m}T,$$

where

(200) $${\left.b_{k,j}^{\left(m\right)}\;\;=\;\;\frac{d^{m}u_{j}\left(x\right)}{dx^{m}}\right|}_{x=x_{k}}.$$

The ***differentiation matrix*** $D^{m}$ contains the coefficients necessary for calculating the *m*th- derivative at the collocation points, and *T* is the column vector of dimension *N* containing the basis functions.

Usually, the functions are interpolated by Lagrange cardinal basis set of order k−1

(201) $$\psi \left(x\right)\approx P_{N-1}\left(x\right)=\sum_{j=1}^{N}\psi \left(x_{j}\right)L_{j}\left(x\right),$$

where

(202) $$L_{k,\;j}\left(x\right)={\prod_{l=1}^{k}}^{\prime }\left(x-x_{l}\right)/{\prod_{l=1}^{k}}^{\prime }\left(x_{j}-x_{l}\right),\;\;\;j=1,...,k,\;{(}^{\prime })\;\;\mathrm{means}\;l\neq j.$$

It is proved that by increasing the order of Lagrange polynomials, the series converges to the corresponding pseudospectral limits. For uniform equidistant grids, $x_{j}=j\Delta x$, and N=2M+1, it is shown that the pseudospectral limit is a sinc–function

(203) $$\lim_{M\to \infty }L_{j}\left(x\right)=\prod_{k=1}^{\infty }\left(1-\frac{{\left(x-x_{j}\right)}^{2}}{k^{2}}\right)=\frac{\sin[\pi \left(x-x_{j}\right)]}{\pi (x-x_{j})}=\mathrm{sinc}\left[\pi \left(x-x_{j}\right)\right].$$

Also, for periodic functions, Fourier series are associated with the cardinal functions

(204) $$\chi_{j}\left(x\right)=e^{i2\pi jx/L}\;⟶\;u_{j}\left(x\right)=\frac{\sin[N\left(x-x_{j}\right)/2]}{N\sin[\left(x-x_{j}\right)/2]}.$$

A relation of Fourier cardinal functions and sinc functions can be seen by the formula

(205) $$\frac{\sin[N\left(x-x_{j}\right)/2]}{N\sin[\left(x-x_{j}\right)/2]}=\sum_{k=-\infty }^{k=\infty }\mathrm{sinc}\left[\left(x-x_{j}+2\pi k\right)/\Delta x\right].$$

Since sinc functions are the infinite order limit of an equispaced FD (Equation (203)), the correspondence now is that periodically repeated FD stencils will tend to the PS limit of Fourier functions as the number of grid points in the stencil approaches the total number of grid points in one period. Also, because equispaced FD can be considered to be a robust sum acceleration scheme of a sinc function series, we expect the same convergence properties of the FD approximation to the Fourier series as the one we find for radial variables [46].

To solve Hamilton’s and variational differential equations [5], we use methods for solving ordinary differential equations, such as those described in the book of Shampine and Gordon [45]. The algorithms are based on predictor-corrector methods with variable order finite-difference approximations, as well as variable time step and backward difference formulae. The accuracy of solutions is controlled by prespecified values.

The action of the Hamiltonian operator on a wavefunction can also be approximated by high-order finite-difference schemes or pseudospectral methods. We have demonstrated that the algorithm developed by Fornberg [25] to construct Lagrange interpolating polynomials for solving the Schrödinger equation is robust and fast. Applications can be found in references [46,47].

### 4.2. The Hénon–Heiles Model

In this section, we show results that demonstrate the effectiveness of solving the Hamiltonian ODEs encountered in the three principal theories: classical, quantum, and thermodynamic. We use a test model that of Hénon–Heiles [26] and mainly the software POMULT [48]. The Hénon–Heiles potential was initially proposed as a model for studying the dynamics of a galaxy. The potential function introduces cubic nonlinearities, and soon, it became the model of choice for numerically investigating the nonlinear behavior of two degrees of freedom systems.

Numerous articles have been published that explore the phase space structures of this nonlinear dynamical system in detail [49]. The Hamiltonian function for a Hénon–Heiles system is written as

(206) $$H_{d}\left(\sigma^{1},\sigma^{2},\pi_{1},\pi_{2}\right)=\frac{1}{2}\left(\pi_{1}^{2}+\pi_{2}^{2}\right)+\frac{1}{2}\left[{\left(\sigma^{1}\right)}^{2}+{\left(\sigma^{2}\right)}^{2}\right]+\left[{\left(\sigma^{1}\right)}^{2}\sigma^{2}-\frac{1}{3}{\left(\sigma^{2}\right)}^{3}\right].$$

**Figure 5:**
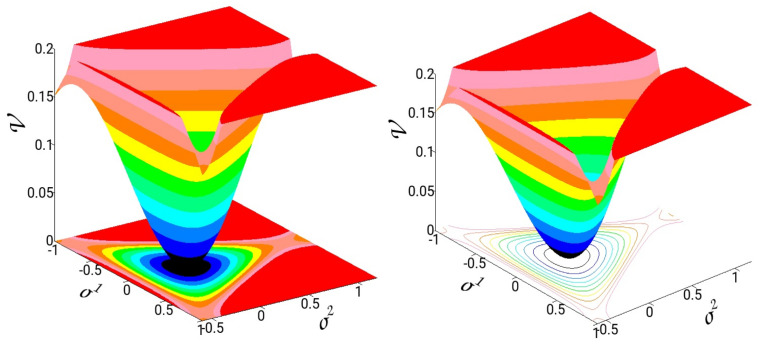
Potential energy surface of the Hénon–Heiles model and isocontours in the configuration plane.

$\left(\sigma^{1},\sigma^{2}\right)$ denote the coordinates and $\left(\pi_{1},\pi_{2}\right)$ the canonical conjugate momenta. In Figure 5, we present 3D graphs and isocontours from the projections of the potential in the coordinate plane. The potential has one minimum at $\left(\sigma^{1},\sigma^{2}\right)=\left(0,0\right)$ and three saddles at the energy of D=1/6. Trajectories with energy above *D* may escape to infinity from the three exit channels. The harmonic normal mode frequencies are $w_{1}=w_{2}=1$, and thus, the system shows a 1:1 resonance. Notice that the spatial symmetry of the Hénon–Heiles potential is $D_{3}$, the same symmetry as triatomic molecules with the same atom.

#### 4.2.1. A Classical Time-Dependent Hénon–Heiles System

A time-dependent Hénon–Heiles system is produced by adding the $q^{0}–$term coupled to $\sigma^{1}–$coordinate (see Figure 1)

(207) $$H\left(q^{0},\sigma^{1},\sigma^{2},\pi_{1},\pi_{2}\right)=H_{d}\left(\sigma^{1},\sigma^{2},\pi_{1},\pi_{2}\right)+A_{f}\sigma^{1}\sin\left(w_{f}q^{0}\right),$$

with $q^{0}=t$, and canonical conjugate momentum, $p_{0}=-H$. We solve Hamilton’s equations as given by Equations (14), and with the extended Hamiltonian, $H_{e}=p_{0}{\dot{q}}^{0}+H=0$.

**Figure 6:**
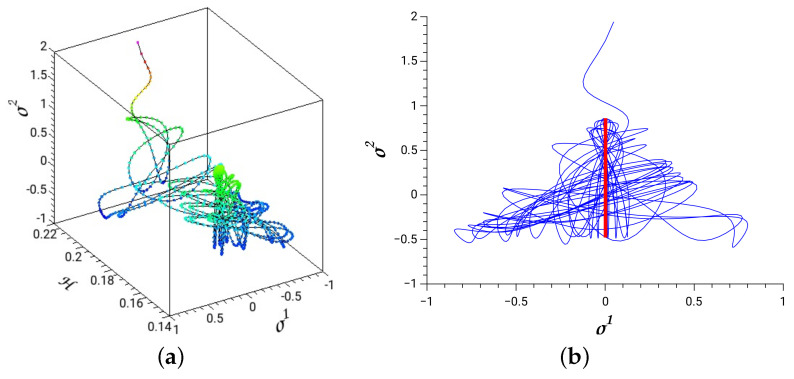
(**a**) A dissociating trajectory of a time-dependent Hénon–Heiles potential. (**b**) The trajectory is initialized from a periodic orbit (red thick line) and with energy $H_{d}=0.1575$.

In Figure 6, we depict a dissociating trajectory with parameters of the time-dependent term, $A_{f}=-0.01$ and $w_{f}=1$. The initial conditions are selected from the stable periodic orbit of the principal family along the $\sigma^{2}$ coordinate and at energy 0.1575. The red thick line in Figure 6b is the projection of this periodic orbit on the coordinate plane. The 3D plot in Figure 6a shows the evolution of the system with the absorbed energy that leads to dissociation.

#### 4.2.2. The Quantum Hénon–Heiles System

We solve the time-dependent Schrödinger equation with an initial Gaussian wavepacket centered at the minimum of the Hénon–Heiles potential, and widths $\Delta \sigma_{1}=0.024$ and $\Delta \sigma_{2}=0.044$. The average energy is 0.124.

The autocorrelation function is the overlap integral of the initial Gaussian with the evolving wavepacket

(208) $$C\left(t\right)=<\psi_{0}|\psi \left(t\right)>.$$

The Fourier transformation of the autocorrelation function depicts the power spectrum from which one can extract eigenenergies and eigenfunctions. Numerical technologies of accurately calculating the energy levels, as well as the corresponding eigenfunctions, have extensively been investigated [50].

**Figure 7:**
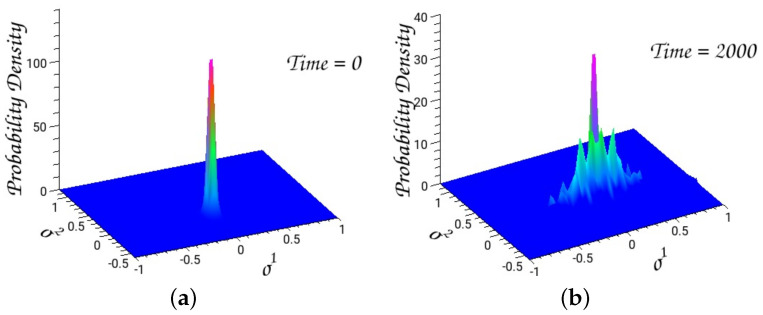
(**a**) The initial wavepacket centered at the minimum of the potential well. (**b**) The evolved wavepacket after 2000 time units.

**Figure 8:**
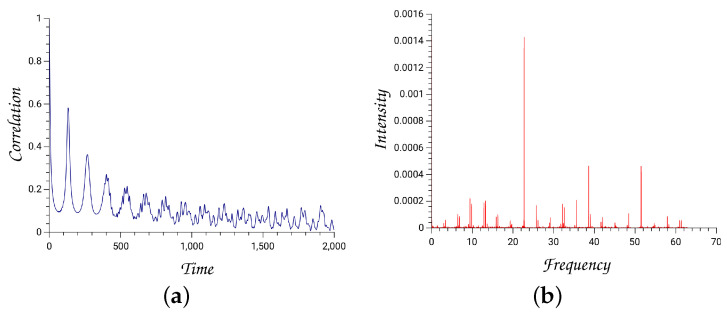
(**a**) The autocorrelation function for the initial Gaussian wavepacket. (**b**) The power spectrum was obtained by taking the Fourier transformation of the autocorrelation function.

The outputs of a typical run are depicted in Figure 7 and Figure 8.

#### 4.2.3. Energy Dissipation of Hénon–Heiles System in Homogeneous Media

For a simple thermodynamic system, such as that of an inert atomic gas with constant volume and particle number (1 mole), the entropy function is given by the equation

(209) $$\Delta S\left(U\right)=c_{V}\ln\left(\frac{U}{U_{0}}\right),$$

with reference the energy $U_{0}$ at T=300K and $c_{V}=\frac{3}{2}N_{A}k_{B}$ to be the specific heat. $k_{B}$ denotes the Boltzmann constant and $N_{A}$ the Avogadro constant.

We assume a diagonal friction parameter matrix $\left[b_{kl}\right]$ that couples the Hénon–Heiles nonlinear oscillator with the homogeneous environment. We assign the nonzero elements $\left(b_{11},b_{22}\right)$ to the values, $\left(b_{11},b_{22}\right)=\left(0.3,0.001\right)$. This is an example of loosely coupling one DOF of the dynamical system to the environment, and we investigate the effect it may have on the entropy production and length of the path [29].

For zero frictions, the dynamical system remains uncoupled to the environment and thus, is a conserved Hamiltonian system. When the friction parameters are turned on, energy dissipates from the dynamical system to the environment. In the extended thermodynamic space, the number of DOF is 6 with coordinates the entropy, *S*, the total energy, *E*, and the four variables of the dynamical system, $\left(\sigma^{1},\sigma^{2},\pi_{1},\pi_{2}\right)$. Hence, the dimension of the extended phase space is 12.

The extended Hamiltonian $H_{e}$ (Equation (162)) is equal to zero on the Lagrangian thermodynamic extended state manifold. We integrate trajectories up to 50 time units with a precision in the value of extended Hamiltonian to about $10^{-15}$.

**Figure 9:**
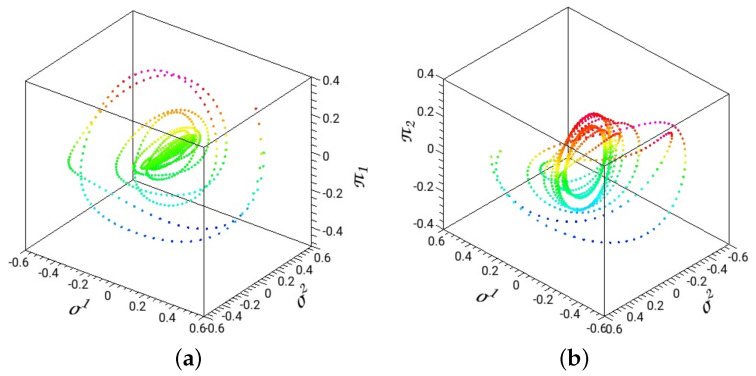
Projections of two representative trajectories in the Hénon–Heiles phase space are shown with friction parameters $b_{11}=0.3\;\mathrm{and}\;b_{22}=0.001$; (**a**) in the $\left(\sigma^{1},\sigma^{2},\pi_{1}\right)$ space and (**b**) in the $\left(\sigma^{1},\sigma^{2},\pi_{2}\right)$ space.

**Figure 10:**
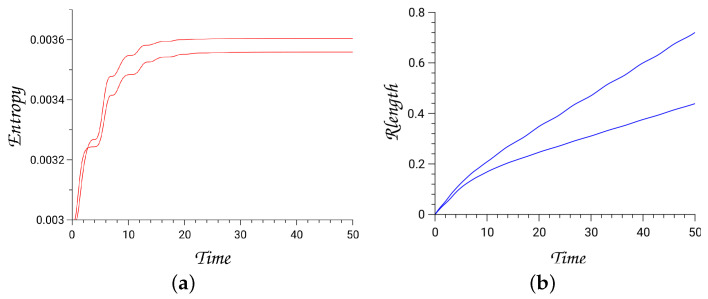
Panel (**a**) is the time evolution of the entropy production and (**b**) the trajectory length calculated with the Ruppeiner metric for the two trajectories shown in Figure 9.

In Figure 9 and Figure 10, the results for two trajectories are depicted by treating the Hénon–Heiles as a dissipating system. We plot the produced entropy (Equation (163)) and the lengths of trajectories, computed via the Ruppeiner metric (Rlength), Equation (154), and with initial energy of the dynamical system $H_{d}=0.125$.

Since one DOF of the dynamical system is weakly coupled to the environment, we find that for the integration times, trajectories are trapped around reduced tori related to the approximately decoupled mode. The thermodynamic length provides an estimate of how close the initial state is to the physical equilibrium state. Hence, we expect trapped trajectories in the approximately decoupled modes to have large lengths and to produce low entropies [29].

## 5. Conclusions

All cardinal physical theories have acquired a Lagrangian or Hamiltonian formalism. The advantages of employing a Hamiltonian framework stem from the fact that global and local constants of motion that dictate the dynamics of the system are respected by solving Hamilton’s equations. In particular, the geometrical structures of Hamiltonian theory were mainly investigated in the second half of the twentieth century with the development of contemporary differential geometry. The purpose of this article is to introduce and unveil common geometrical structures of the three most frequently applied theories in computational chemistry, those of classical mechanics, quantum mechanics, and classical thermodynamics, all of them at the non-relativistic approximation. We have shown that working in extended phase space, the physical states of the system in the three theories are described by Lagrangian submanifolds. Observables are calculated by canonically projecting the extended phase space in a reduced dimensional space. In classical mechanics, integrable systems guarantee the existence of n+1 constants of motion, and thus (n+1)–dimensional Lagrangian submanifolds embedded into 2(n+1)–dimensional phase space. Quantum systems can also be considered to be integrable systems in the projective Hilbert space. Finally, classical thermodynamics have also been given a Hamiltonian formalism in an extended phase space, similar to classical mechanics, and capable of formulating irreversible processes. We can obtain representations of Lagrangian manifolds when we use Hamiltonians in action-angle variables in classical mechanics and Gibbs’s fundamental equation in classical thermodynamics.

Noether’s theorem [51] proves that constants of motion are associated with symmetries of the system, which leave the Hamiltonian function invariant. Global continuous symmetries, such as time-reversal, translational, and rotational transformations, are theorized by Lie groups and their associated Lie algebras. Advanced methods to extract the constants of motion related to symmetries and to reduce the dimensionality of the problem have been developed in recent decades [2].

Here, we have not explored the important role of symmetry in dynamical systems. However, we have emphasized the importance of approximating local constants of motion by solving the variational equations of a Hamiltonian system, such as around equilibria and stable periodic orbits. This requires the diagonalization of the fundamental matrix (see Section 2.1.5). It is proved that each constant of motion corresponds to a pair of eigenvalues equal to one.

Numerically solving the equations of motion (classical, quantum, thermodynamic) is the main task of computational chemistry. Finite-difference methods have been broadly adopted for finding solutions of ordinary differential equations, as well as partial differential equations, such as the Schrödinger equation. Nevertheless, in spite of their many successes, they are restricted to low-dimensional systems since they require substantial computational resources for many degrees of freedom systems. Furthermore, non-symplectic integrators for Hamiltonian ODEs inevitably introduce numerical instability in long time integrations.

Molecules are generally complex systems with many degrees of freedom. Their phase space is entangled with regular and chaotic regions. Therefore, it is not a surprise that computational chemistry has always tried to exploit new computer advances, such as parallel computing (including grid and cloud), as well as GPU technology. As far as the integration of the trajectories of large molecules for a long time is concerned, the good performance of several symplectic integrators has been recognized. Among the most popular ones are the simple low-order symplectic algorithms, such as the leapfrog or velocity Verlet algorithms [52].

Presently, AI (artificial intelligence) methods, particularly machine-learning techniques, are under exploration. In the last few years, an intense interest has been directed at finding algorithms that incorporate physics knowledge into neural networks. Hamiltonian neural networks label techniques that try to solve Hamilton’s equations with deep neural networks [53,54,55]. Solving the Schrödinger equation has also attracted the interest of researchers in this field [56]. However, we must note that at present, physics neural networks (PNN) algorithms are tested with low-dimensional systems, and their extension to systems with many degrees of freedom remains to be proved.

Irrespective of the final result of this endeavor, what is worth pursuing is developing computational methods that take into account multiscale physical theories that cover extended temporal/spatial scales. What we have shown in this review is that Hamiltonian (symplectic) geometry is the foundation of principal physical theories. This should be taken into account in building PNN algorithms [57], and it signals the need for new projects. Hamiltonian geometry yields the necessary and sufficient conditions for the mutual assistance of humans and machines in deep-learning processes.

## Figures and Tables

**Figure A1:**
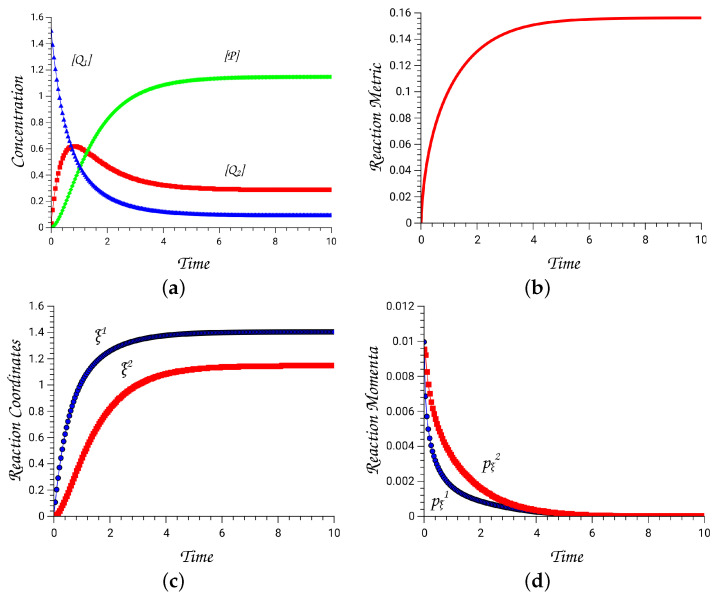
(**a**) Concentrations of the constituent chemical species as functions of time, (**b**) reaction metric, (**c**) reaction coordinates $\left(\xi^{1},\xi^{2}\right)$, (**d**) conjugate momenta $\left(p_{\xi^{1}},p_{\xi^{2}}\right)$ to reaction coordinates. The quantities have been calculated from a trajectory run with initial concentrations, $[Q_{1}]=1.5,$ $[Q_{2}]=0.03,$ [P]=0.001. The rate constants for the forward reactions are taken equal to $k_{f1}=1.5,$ $k_{f2}=1.0$, and the backward reactions $k_{b1}=0.5,\;k_{b2}=0.25$.

## Data Availability

The raw data supporting the conclusions of this article will be made available by the authors on request.

## Abbreviations

The following abbreviations are used in this manuscript:

FD	Finite Difference
PS	Pseudospectral
DOF	degrees of freedom
TEPS	Thermodynamic Extended Phase Space
TEPSS	Thermodynamic Extended Physical State Submanifold
TCS	Thermodynamic Contact Space
PTS	Physical Thermodynamic Submanifold
ODEs	Ordinary Differential Equations
HNN	Hamiltonian neural networks
PNN	Physics neural networks
nD	n–dimensional

## Appendix A

### Appendix A.1. Proof of Equation (89)

For an observable $\hat{O}$ and normalized states, <ψ|ψ>=1, its expectation value is the function

$$O:H_{r}^{2n}\mapsto R,$$

and

$$O=<\psi |\hat{O}|\psi >=\frac{1}{2\hbar }G(|\psi >,\hat{O}\left|\psi >\right).$$

We can prove that the differential 1–form of the expectation value in the real projective Hilbert space is given by

(A1) $$dO\left(|\phi >\right)=i_{X_{\hat{O}}}\Omega \left(|\phi >\right)=\Omega \left(X_{\hat{O}},|\phi >\right),$$

where $|\phi >\;\in \;T_{|\psi_{P}>}P^{n}\left(H^{n+1}\right)$ and normalized states <ψ|ψ>=1.

Indeed,

(A2) $$\begin{array}{ccc} dO(|\phi >) & = & L_{X_{\hat{O}}}|\phi >=\frac{\partial }{\partial \eta }<\psi +\eta \phi |\hat{O}{\left|\psi +\eta \phi >\right|}_{\eta =0}\\ & = & <\phi |\hat{O}\left|\psi >+<\psi \right|\hat{O}\left|\phi >=<\phi \right|\hat{O}\left|\psi >+<\phi \right|{\hat{O}}^{†}|\psi >\\ & = & 2<\phi |\hat{O}|\psi >\\ & = & \frac{1}{\hbar }G(|\phi >,\hat{O}\left|\psi >\right)=-\frac{1}{\hbar }\Omega (|\phi >,J\hat{O}\left|\psi >\right)\left(Equation\;\left(83\right)\right)\\ & = & \Omega \left(|\phi >,\left(-\frac{J}{\hbar }\hat{O}\right)|\psi >\right)=\Omega \left(|\phi >,\left(\frac{𝚤}{\hbar }\hat{O}\right)|\psi >\right)\\ & = & \Omega (|\phi >,-X_{\hat{O}}|\psi >)=\Omega (X_{\hat{O}}\left|\psi >,|\phi >\right)\\ & = & i_{X_{\hat{O}}}\Omega \left(|\phi >\right). \end{array}$$

According to Equation (15) we have replaced −J with 𝚤.

### Appendix A.2. Proof of Equation (90)

If $\left(O_{1},O_{2}\right)$ are the expectation value functions of two observables, the Poisson bracket is defined by the equation

(A3) $$\left\{O_{1},O_{2}\right\}=\Omega \left(X_{{\hat{O}}_{1}},X_{{\hat{O}}_{2}}\right)=<\psi \left|-\frac{𝚤}{\hbar }\left[{\hat{O}}_{1},{\hat{O}}_{2}\right]\right|\psi >.$$

$\left[{\hat{O}}_{1},{\hat{O}}_{2}\right]$ is the commutator of the two operators $\left({\hat{O}}_{1},{\hat{O}}_{2}\right)$. Indeed, the Poisson bracket of the expectation functions $\left(O_{1},O_{2}\right)$ at state |ψ> is developed as follows

(A4) $$\begin{array}{ccc} \left\{O_{1},O_{2}\right\} & = & \Omega (X_{{\hat{O}}_{1}},X_{{\hat{O}}_{2}})|\psi >\\ & = & X_{{\hat{O}}_{1}}|\psi >X_{{\hat{O}}_{2}}|\psi >-X_{{\hat{O}}_{2}}\left|\psi >X_{{\hat{O}}_{1}}\right|\psi >\\ & = & <\psi |X_{{\hat{O}}_{1}}X_{{\hat{O}}_{2}}\left|\psi >-<\psi \right|X_{{\hat{O}}_{2}}\left|X_{{\hat{O}}_{1}}\right|\psi >\\ & = & <\psi |\frac{-𝚤}{\hbar }\left({\hat{O}}_{1}{\hat{O}}_{2}-{\hat{O}}_{2}{\hat{O}}_{1}\right)\left|\psi >\right)\\ & = & <\psi |\frac{-𝚤}{\hbar }\left[{\hat{O}}_{1},{\hat{O}}_{2}\right]|\psi >. \end{array}$$

### Appendix A.3. Proof of Equation (152)

For a thermodynamic system the Hamiltonian in the extended phase space is written as, $H_{e}\left(q^{0},q^{1},\ldots ,q^{n},p_{0},p_{1},\ldots ,p_{n}\right)=-p_{0}F\left(q^{0},q^{1},\ldots ,q^{n},\gamma_{1},\ldots ,\gamma_{n}\right),\mathrm{where}\gamma_{i}=-\frac{p_{i}}{p_{0}}$. Then, the time derivative of an observable function O(q,p) is given by the Poisson brackets as

(A5) $$\begin{array}{ccc} \dot{O} & = & \left\{O,H_{e}\right\}\\ & = & \sum_{i=0}^{n}\left[\frac{\partial O}{\partial q^{i}}\frac{\partial H_{e}}{\partial p_{i}}-\frac{\partial O}{\partial p_{i}}\frac{\partial H_{e}}{\partial q^{i}}\right]\\ & = & \left(\frac{\partial O}{\partial q^{0}}\frac{\partial H_{e}}{\partial p_{0}}-\frac{\partial O}{\partial p_{0}}\frac{\partial H_{e}}{\partial q^{0}}\right)+\sum_{i=1}^{n}\left[\frac{\partial O}{\partial q^{i}}\frac{\partial H_{e}}{\partial p_{i}}-\frac{\partial O}{\partial p_{i}}\frac{\partial H_{e}}{\partial q^{i}}\right]\\ & = & \left(\frac{\partial O}{\partial q^{0}}{\dot{q}}^{0}+\frac{\partial O}{\partial p_{0}}{\dot{p}}_{0}\right)+\sum_{i=1}^{n}\left[\frac{\partial O}{\partial q^{i}}{\dot{q}}^{i}+\frac{\partial O}{\partial p_{i}}{\dot{p}}_{i}\right]. \end{array}$$

The partial derivatives are

$$\begin{array}{ccc} \frac{\partial O}{\partial p_{0}} & = & \sum_{i=1}^{n}\frac{\partial O}{\partial \gamma_{i}}\left(\frac{p_{i}}{p_{0}^{2}}\right)=-\frac{1}{p_{0}}\sum_{i=1}^{n}\frac{\partial O}{\partial \gamma_{i}}\gamma_{i}\\ \frac{\partial O}{\partial p_{i}} & = & \frac{\partial O}{\partial \gamma_{i}}\left(-\frac{1}{p_{0}}\right)=-\frac{1}{p_{0}}\frac{\partial O}{\partial \gamma_{i}},\;\;i=1,\ldots ,n\\ \frac{\partial H_{e}}{\partial p_{0}} & = & -F-p_{0}\sum_{i=1}^{n}\frac{\partial F}{\partial \gamma_{i}}\left(\frac{p_{i}}{p_{0}^{2}}\right)=-F+\sum_{i=1}^{n}\frac{\partial F}{\partial \gamma_{i}}\gamma_{i}\\ \frac{\partial H_{e}}{\partial p_{i}} & = & -p_{0}\frac{\partial F}{\partial \gamma_{i}}\left(-\frac{1}{p_{0}}\right)=\frac{\partial F}{\partial \gamma_{i}},\;\;i=1,\ldots ,n\\ \frac{\partial H_{e}}{\partial q^{0}} & = & -p_{0}\frac{\partial F}{\partial q^{0}}\\ \frac{\partial H_{e}}{\partial q^{i}} & = & -p_{0}\frac{\partial F}{\partial q^{i}},\;\;i=1,\ldots ,n, \end{array}$$

that result in

$$\begin{array}{ccc} \dot{O} & = & \frac{\partial O}{\partial q^{0}}\left(-F+\sum_{i=1}^{n}\gamma_{i}\frac{\partial F}{\partial \gamma_{i}}\right)-\sum_{i=1}^{n}\frac{\partial O}{\partial \gamma_{i}}\gamma_{i}\frac{\partial F}{\partial q^{0}}+\sum_{i=1}^{n}\left[\frac{\partial O}{\partial q^{i}}\frac{\partial F}{\partial \gamma_{i}}-\left(-\frac{1}{p_{0}}\frac{\partial O}{\partial \gamma_{i}}\right)\left(-p_{0}\frac{\partial F}{\partial q^{i}}\right)\right]\\ & = & \frac{\partial O}{\partial q^{0}}\left(-F+\sum_{i=1}^{n}\gamma_{i}\frac{\partial F}{\partial \gamma_{i}}\right)-\sum_{i=1}^{n}\frac{\partial O}{\partial \gamma_{i}}\gamma_{i}\frac{\partial F}{\partial q^{0}}+\sum_{i=1}^{n}\frac{\partial O}{\partial q^{i}}\frac{\partial F}{\partial \gamma_{i}}-\sum_{i=1}^{n}\frac{\partial O}{\partial \gamma_{i}}\frac{\partial F}{\partial q^{i}}\\ & = & \frac{\partial O}{\partial q^{0}}\left(-F+\sum_{i=1}^{n}\gamma_{i}\frac{\partial F}{\partial \gamma_{i}}\right)+\sum_{i=1}^{n}\frac{\partial O}{\partial q^{i}}\frac{\partial F}{\partial \gamma_{i}}-\sum_{i=1}^{n}\frac{\partial O}{\partial \gamma_{i}}\left(\frac{\partial F}{\partial q^{i}}+\gamma_{i}\frac{\partial F}{\partial q^{0}}\right). \end{array}$$

Hence, the ***thermodynamic contact vector field*** is

(A6) $$\begin{array}{ccc} \dot{q^{0}} & = & X_{c}^{0}=-F+\sum_{i=1}^{n}\gamma_{i}\frac{\partial F}{\partial \gamma_{i}} \end{array}$$

(A7) $$\begin{array}{ccc} \dot{q^{i}} & = & X_{c}^{i}=\frac{\partial F}{\partial \gamma_{i}},\;\;\;\;i=1,\ldots ,n \end{array}$$

(A8) $$\begin{array}{ccc} {\dot{\gamma }}_{i} & = & {\bar{X}}_{c}^{i}=-\left(\frac{\partial F}{\partial q^{i}}+\gamma_{i}\frac{\partial F}{\partial q^{0}}\right),\;\;\;\;i=1,\ldots ,n. \end{array}$$

### Appendix A.4. Tables

#### Appendix A.4.1. Projection Maps between Thermodynamic Extended Phase Space and Thermodynamic Contact Space

**Table A1:** Projection maps (π) between thermodynamic extended phase space (TEPS) and thermodynamic contact space (TCS). $\pi^{-1}$ denotes the inverse map, $\pi_{*}$ the push-forward of vector fields operation and $\pi^{*}$ the pull-back of functions (0–forms). $P^{2n+2}$ is the extended phase space, $C^{2n+1}$ the contact state space, $L_{p}^{n+1}$ the Lagrangian submanifold in TEPS, which describes the thermodynamic extended physical state submanifold (TEPSS), $L_{c}^{n}$ the Legendrian submanifold in TCS, which describes the physical thermodynamic submanifold (PTS), $H_{e}$ the extended Hamiltonian, $X_{H_{e}}$ the extended Hamiltonian vector field, $H_{c}$ the contact Hamiltonian and $X_{H_{c}}$ the contact Hamiltonian vector field.

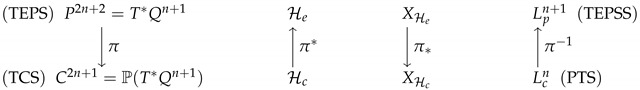

#### Appendix A.4.2. Thermodynamic Manifolds in Entropy Representation

**Table A2:** Thermodynamic Manifolds in Entropy Representation.

Manifold	$P^{2n}\equiv T^{*}Q^{n}$	$C^{2n+1}\equiv P\left(T^{*}Q^{n+1}\right)$	$P^{2n+2}\equiv T^{*}Q^{n+1}$
Coordinates			
n=2+r	$q^{1}=U,q^{2}=V,$	$q^{0}=S,q^{1}=U,$	$q^{0}=S,q^{1}=U,$
	$q^{k}=N^{k-2}$	$q^{2}=V,q^{k}=N^{k-2}$	$q^{2}=V,q^{k}=N^{k-2}$
Momenta			
$p_{0}=-1$	$\gamma_{1}=\frac{1}{T}$	$\gamma_{1}=\frac{1}{T}$	$p_{1}=-p_{0}\gamma_{1}$
	$\gamma_{2}=\frac{P}{T}$	$\gamma_{2}=\frac{P}{T}$	$p_{2}=-p_{0}\gamma_{2}$
	$\gamma_{k}=-\frac{\mu_{k-2}}{T}$	$\gamma_{k}=-\frac{\mu_{k-2}}{T}$	$p_{k}=-p_{0}\gamma_{k}$
1–form			
	$\theta =\sum_{i=1}^{n}\gamma_{i}dq^{i}$	$\theta_{c}=dq^{0}-\sum_{i=1}^{n}\gamma_{i}dq^{i}$	$\theta_{e}=\sum_{i=0}^{n}p_{i}dq^{i}$
2–form			
ω=−dθ	$\omega =\sum_{i=1}^{n}dq^{i}\wedge d\gamma_{i}$		$\omega_{e}=\sum_{i=0}^{n}dq^{i}\wedge dp^{i}$
PTS	$L_{p}^{n}$	$L_{c}^{n}$	$L_{p}^{n+1}$ (TEPSS)
	ω=0,H=S	$\theta_{c}=0,\;\;F=S$	$\theta_{e}=0,\;\;\omega_{e}=0$
			$H_{e}=0,\;\;L_{X_{H_{e}}}\theta_{e}=0$
Metric	$dl^{2}=-\sum_{i=1}^{n}dq^{i}⊗d\gamma_{i}$	$dl^{2}=-\sum_{i=1}^{n}dq^{i}⊗d\gamma_{i}$	$dl^{2}=-\sum_{i=1}^{n}dq^{i}⊗d\gamma_{i}$

#### Appendix A.4.3. Thermodynamic Manifolds in Energy Representation

**Table A3:** Thermodynamic Manifolds in Energy Representation.

Manifold	$P^{2n}\equiv T^{*}Q^{n}$	$C^{2n+1}\equiv P\left(T^{*}Q^{n+1}\right)$	$P^{2n+2}\equiv T^{*}Q^{n+1}$
Coordinates			
n=2+r	$q^{0}=S,q^{2}=V$	$q^{0}=S,q^{1}=U$	$q^{0}=S,q^{1}=U$
	$q^{k}=N^{k-2}$	$q^{2}=V,q^{k}=N^{k-2}$	$q^{2}=V,q^{k}=N^{k-2}$
Momenta			
$p_{1}=-1$	$\beta_{0}=T$	$\beta_{0}=T$	$p_{0}=-p_{1}\beta_{0}$
	$\beta_{2}=-P$	$\beta_{2}=-P$	$p_{2}=-p_{1}\beta_{2}$
	$\beta_{k}=\mu_{k-2}$	$\beta_{k}=\mu_{k-2}$	$p_{k}=-p_{1}\beta_{k}$
1–form			
	$\theta =\beta_{0}dq^{0}+\sum_{i=2}^{n}\beta_{i}dq^{i}$	$\theta_{c}=dq^{1}-\beta_{0}dq^{0}-$	$\theta_{e}=\sum_{i=0}^{n}p_{i}dq^{i}$
		$\sum_{i=2}^{n}\beta_{i}dq^{i}$	
2–form			
ω=−dθ	$\omega =dq^{0}\wedge d\beta_{0}+\sum_{i=2}^{n}dq^{i}\wedge d\beta_{i}$		$\omega_{e}=\sum_{i=0}^{n}dq^{i}\wedge dp^{i}$
PTS	$L_{p}^{n}$	$L_{c}^{n}$	$L_{p}^{n+1}$ (TEPSS)
	ω=0,H=U	$\theta_{c}=0,\;\;F=U$	$\theta_{e}=0,\;\;\omega_{e}=0$
			$H_{e}=0,\;\;L_{X_{H_{e}}}\theta_{e}=0$
Metric	$dl^{2}=dq^{0}⊗d\beta_{0}+$	$dl^{2}=dq^{0}⊗d\beta_{0}+$	$dl^{2}=dq^{0}⊗d\beta_{0}+$
	$\sum_{i=2}^{n}dq^{i}⊗d\beta_{i}$	$\sum_{i=2}^{n}dq^{i}⊗d\beta_{i}$	$\sum_{i=2}^{n}dq^{i}⊗d\beta_{i}$

### Appendix A.5. Hamiltonian Chemical Kinetics: A Simple Chemical Kinetic Example: Consecutive First-Order Elementary Reactions

Here, we present how a classical kinetic network can be cast into Hamiltonian formalism. As an example, we take two consecutive elementary chemical reactions and dress them with a Hamiltonian mantle [30]. Our generalized coordinates now, instead of distances and angles to define the positions of the atoms in the molecule, as a microscopic description requires, are the macroscopic extensive properties, such as internal energy, volume, and the number of moles (or molecules). The conjugate momenta to these thermodynamic coordinates are related to the intensive properties of temperature, pressure, and chemical potentials, respectively. By treating the macroscopic system in phase space and integrating Hamilton’s equations, we can define measures for classifying the trajectories and investigate alternative kinetic models.

The chemical equations are

$$\begin{array}{ccc} Q_{1} & \underset{k_{b1}}{\overset{k_{f1}}{⇌}} & Q_{2}\\ Q_{2} & \underset{k_{b2}}{\overset{k_{f2}}{⇌}} & P, \end{array}$$

where $\left(k_{f1},k_{f2}\right)$ are the rate constants for the forward reactions and $\left(k_{b1},k_{b2}\right)$ the rate constants for the backward reactions. $Q_{1}$, $Q_{2}$ and P are the constituent compounds with concentrations

(A9) $$x_{1}=\left[Q_{1}\right],\;\;\;\;x_{2}=\left[Q_{2}\right],\;\;\;\;x_{3}=\left[P\right].$$

The reaction coordinates (extension or progress of the reactions) are denoted by $\xi ={\left(\xi^{1},\xi^{2}\right)}^{T}$. Then, the time variation of concentrations is written as

(A10) $$\left(\begin{array}{c} {\dot{x}}_{1}\\ {\dot{x}}_{2}\\ {\dot{x}}_{3} \end{array}\right)=C\dot{\xi }\left(t\right)=\left(\begin{array}{cc} -1 & 0\\ 1 & -1\\ 0 & 1 \end{array}\right)\left(\begin{array}{c} {\dot{\xi }}^{1}\\ {\dot{\xi }}^{2} \end{array}\right).$$

*C* is the stoichiometric matrix.

We consider the reactive system as a closed thermodynamic system, i.e., it exchanges energy with the environment, but not mass. If the initial concentrations of the three substances $Q_{1}$, $Q_{2}$ and P are $x_{1}\left(0\right)=x_{10},\;\;x_{2}\left(0\right)=x_{20}$ and $x_{3}\left(0\right)=x_{30}$, respectively, and assuming ξ=0 at t=0, then the solution of Equations (A10) is

(A11) $$\begin{array}{ccc} x_{1}\left(t\right) & = & x_{10}-\xi^{1}\left(t\right)\\ x_{2}\left(t\right) & = & x_{20}+\xi^{1}\left(t\right)-\xi^{2}\left(t\right)\\ x_{3}\left(t\right) & = & x_{30}+\xi^{2}\left(t\right). \end{array}$$

According to the law of mass action [37,38,39,40] the reaction rates are written as

$$\begin{array}{ccc} R_{f1} & = & k_{f1}x_{1}\\ R_{b1} & = & k_{b1}x_{2}\\ R_{f2} & = & k_{f2}x_{2}\\ R_{b2} & = & k_{b2}x_{3}, \end{array}$$

where $\left(R_{f1},R_{f2}\right)$ are the rates of the forward reactions and $\left(R_{b1},R_{b2}\right)$ the rates of the backward reactions, respectively. Then, the rates of reaction coordinates are

(A12) $$\begin{array}{ccc} {\dot{\xi }}^{1} & = & R_{f1}-R_{b1}=k_{f1}x_{1}-k_{b1}x_{2}\\ & = & k_{f1}\left(x_{10}-\xi^{1}\right)-k_{b1}\left(x_{20}+\xi^{1}-\xi^{2}\right)\\ {\dot{\xi }}^{2} & = & R_{f2}-R_{b2}=k_{f2}x_{2}-k_{b2}x_{3}\\ & = & k_{f2}\left(x_{20}+\xi^{1}-\xi^{2}\right)-k_{b2}\left(x_{30}+\xi^{2}\right). \end{array}$$

The analytical solutions of the above differential equations for the case $k_{b1}=k_{b2}=0$, as well as $x_{20}=x_{30}=0$ are

$$\begin{array}{ccc} x_{1}\left(t\right) & = & x_{10}\exp\left(-k_{f1}t\right)\\ x_{2}\left(t\right) & = & x_{10}\left(\frac{k_{f1}}{k_{f2}-k_{f1}}\right)\left[\exp\left(-k_{f1}t\right)-\exp\left(-k_{f2}t\right)\right]\\ x_{3}\left(t\right) & = & x_{10}\left(\frac{1}{k_{f2}-k_{f1}}\right)\left[k_{f2}\left(1-\exp(-k_{f1}t)\right)-k_{f1}\left(1-\exp(-k_{f2}t\right)\right], \end{array}$$

with limits at equilibrium (t→∞)

$$x_{1}^{eq}=x_{2}^{eq}=0\;\;\;\;\;x_{3}^{eq}=x_{10}.$$

As discussed in Section 3, the extended phase space is obtained by adding the entropy to the set of generalized coordinates and $p_{0}$ as a conjugate momentum. Then, the momenta of thermodynamic coordinates are defined as $p=p_{0}\mu$.

We study chemical reactions under isothermal and isobaric conditions. In this case, we take $p_{0}$ to denote the conjugate momentum of the entropy expressed by the Massieu-Gibbs function, S=−G/T, with *G* to be the Gibbs thermodynamic potential. The Affinities of the reactions, $A_{\xi^{k}}$, are then written as

(A13) $$A_{\xi^{k}}=RT\ln\left(\frac{R_{fk}\left(\xi \right)}{R_{bk}\left(\xi \right)}\right),\;\;\;\;k=1,2.$$

Following the theory described in Section 3.4.2, the above kinetic scheme is put in a Hamiltonian framework by first writing the Hamiltonian in reaction coordinates

(A14) $$H_{\xi }^{S}=\sum_{k=1}^{2}\left(p_{\xi^{k}}+\frac{p_{0}}{T}A_{\xi^{k}}\right){\dot{\xi }}^{k},$$

where $\left(p_{\xi^{1}},p_{\xi^{2}}\right)$ denote the conjugate momenta of ${\left(\xi^{1},\xi^{2}\right)}^{T}$. The reaction affinities are written as

(A15) $$A_{\xi^{1}}=RT\ln\left(\frac{k_{f1}\left(x_{10}-\xi^{1}\right)}{k_{b1}\left(x_{20}+\xi^{1}-\xi^{2}\right)}\right).$$

(A16) $$A_{\xi^{2}}=RT\ln\left(\frac{k_{f2}\left(x_{20}+\xi^{1}-\xi^{2}\right)}{k_{b2}\left(x_{30}+\xi^{2}\right)}\right).$$

Hence, Hamilton’s equations take the form (Equation (192))

(A17) $$\begin{array}{ccc} \dot{S} & = & \frac{\partial H_{\xi }^{S}}{\partial p_{0}}=\frac{A_{\xi^{1}}}{T}{\dot{\xi }}^{1}+\frac{A_{\xi^{2}}}{T}{\dot{\xi }}^{2} \end{array}$$

(A18) $$\begin{array}{ccc} {\dot{\xi }}^{1} & = & \frac{\partial H_{\xi }^{S}}{\partial p_{\xi^{1}}}=k_{f1}\left(x_{10}-\xi^{1}\right)-k_{b1}\left(x_{20}+\xi^{1}-\xi^{2}\right) \end{array}$$

(A19) $$\begin{array}{ccc} {\dot{\xi }}^{2} & = & \frac{\partial H_{\xi }^{S}}{\partial p_{\xi^{2}}}=k_{f2}\left(x_{20}+\xi^{1}-\xi^{2}\right)-k_{b2}\left(x_{30}+\xi^{2}\right) \end{array}$$

(A20) $$\begin{array}{ccc} {\dot{p}}_{0} & = & -\frac{\partial H_{\xi }^{S}}{\partial S}=0 \end{array}$$

(A21) $$\begin{array}{ccc} {\dot{p}}_{\xi^{1}} & = & -\frac{\partial H_{\xi }^{S}}{\partial \xi^{1}}=-\frac{p_{0}}{T}\sum_{i=1}^{2}\left(\frac{\partial A_{\xi^{i}}}{\partial \xi^{1}}\right){\dot{\xi }}^{i}-\sum_{i=1}^{2}\left(p_{\xi^{i}}+\frac{p_{0}}{T}A_{\xi^{i}}\right)\frac{\partial {\dot{\xi }}^{i}}{\partial \xi^{1}} \end{array}$$

(A22) $$\begin{array}{ccc} {\dot{p}}_{\xi^{2}} & = & -\frac{\partial H_{\xi }^{S}}{\partial \xi^{2}}=-\frac{p_{0}}{T}\sum_{i=1}^{2}\left(\frac{\partial A_{\xi^{i}}}{\partial \xi^{2}}\right){\dot{\xi }}^{i}-\sum_{i=1}^{2}\left(p_{\xi^{i}}+\frac{p_{0}}{T}A_{\xi^{i}}\right)\frac{\partial {\dot{\xi }}^{i}}{\partial \xi^{2}}. \end{array}$$

#### Appendix A.5.1. Discussion

We make the following remarks. Integrating Equation (A17), we calculate the entropy production for the selected reaction pathway. It also provides the time variation of Gibbs free energy ($\dot{G}=-T\dot{S}$). Equations (A18) and (A19) are the fluxes of chemical reactions given as the difference of the rates of forward and backward reactions. Usually, reaction rates are obtained by phenomenological kinetic models, such as those provided by the Mass Action kinetics (MAK) [37,38].

Equation (A20) designates the conservation of the gauge momentum $p_{0}$ with an initial value equal to −1.

The terms in the second summation of the RHS of Equations (A21) and (A22) are zero on the thermodynamic extended state manifold (Lagrangian submanifold), and they can be ignored during the integration of Hamilton’s equations. However, in solving the variational equations, we must keep all the terms.

By studying the kinetics of chemical reactions in phase space, we can adopt measures for classifying reaction paths in phase space. For example, we define a metric on the Lagrangian submanifold in Massieu-Gibbs representation as

(A23) $$dl^{2}=\frac{1}{p_{0}}\sum_{i=1}^{2}dp_{\xi^{i}}d\xi^{i}=-\sum_{i=1}^{2}\sum_{k=1}^{2}\frac{\partial^{2}S}{\partial \xi^{i}\partial \xi^{k}}d\xi^{i}d\xi^{k}=\frac{1}{T}\sum_{i=1}^{2}\sum_{k=1}^{2}\frac{\partial^{2}G}{\partial \xi^{i}\partial \xi^{k}}d\xi^{i}d\xi^{k},$$

and calculate the distance between the initial and equilibrium states

(A24) $$L\left(t_{max}\right)=\int_{0}^{t_{max}}\sqrt{-\sum_{i=1}^{2}{\dot{p}}_{\xi^{i}}{\dot{\xi }}^{i}}\;\;dt,$$

with $t_{max}$ to be the maximum integration time and substituting $p_{0}=-1$. The distance of two states has been utilized to define a better low bound of the entropy production or dissipated work (availability) than zero for finite time irreversible processes [43,44].

For the chosen example, we have numerically solved Hamilton’s equations for several combinations of rate constants and initial concentrations [30]. A representative trajectory is shown in Figure A1 with parameter values given in the caption of the figure. For all trajectories run with double precision arithmetic, the extended Hamiltonian $H_{\xi }^{S}$ is conserved approximately to zero with an accuracy $\approx 10^{-15}$. The symplectic fundamental matrix is obtained by solving the variational equations [29,48] for a time interval of 5 t.u. (time units). Diagonalizing this matrix, we find three pairs of eigenvalues. One pair is always equal to one as a result of the conservation of the Hamiltonian function, whereas the other two come in combinations of two real positive numbers $\left(\sigma_{i},\frac{1}{\sigma_{i}}\right)$. For the present trajectory, these are $(0.18478\times 10^{6},0.54117\times 10^{-5)}$ and (61.74712,0.016195). All panels in Figure A1 demonstrate that for this highly unstable trajectory, equilibrium is reached in 4 t.u., with the momenta to approach zero.
